# Intrauterine botulinum toxin A administration promotes endometrial regeneration mediated by IGFBP3-dependent OPN proteolytic cleavage in thin endometrium

**DOI:** 10.1007/s00018-022-04684-6

**Published:** 2023-01-05

**Authors:** Danbi Lee, Jungho Ahn, Hwa Seon Koo, Youn-Jung Kang

**Affiliations:** 1 Department of Biomedical Science, School of Life Science, CHA University, 335 Pangyo-ro, Bundang-gu, Seongnam-si, Gyeonggi-do South Korea; 2 Department of Biochemistry, School of Medicine, Research Institute for Basic Medical Science, CHA University, 335 Pangyo-ro, Bundang-gu, Seongnam-si, Gyeonggi-do South Korea; 3 CHA Fertility Center Bundang, 59, Yatap-ro, Bundang-gu, Seongnam-si, Gyeonggi-do South Korea

**Keywords:** Thin endometrium, Endometrial regeneration, Botulinum toxin A, Insulin-like growth factor binding protein-3, Osteopontin

## Abstract

Adequate endometrial growth is a critical factor for successful embryo implantation and pregnancy maintenance. We previously reported the efficacy of intrauterine administration of botulinum toxin A (BoTA) in improving the endometrial angiogenesis and the rates of embryo implantation. Here, we further evaluated its potent therapeutic effects on the uterine structural and functional repair and elucidated underlying molecular regulatory mechanisms. This study demonstrated that a murine model of thin endometrium was successfully established by displaying dramatically decreased endometrial thickness and the rates of embryo implantation compared to normal endometrium. Interestingly, the expressions of insulin-like growth factor binding protein-3 (IGFBP3) and an active 35 kDa-form of osteopontin (OPN) were significantly reduced in thin endometrium, which were almost fully restored by intrauterine BoTA administration. Neutralization of BoTA-induced IGFBP3 subsequently suppressed proteolytic cleavage of OPN, exhibiting un-recovered endometrial thickness even in the presence of BoTA administration, suggesting that BoTA-induced endometrial regeneration might be mediated by IGFBP3-dependent OPN proteolytic cleavage. Our findings suggest that intrauterine BoTA administration improves the endometrial environment in our murine model with thin endometrium by increasing endometrial receptivity and angiogenesis in a manner dependent on the regulatory effect of IGFBP3 on OPN proteolytic cleavage, proposing BoTA as an efficient therapeutic strategy for the patients with thin endometrium.

**Supplementary Information:**

The online version contains supplementary material available at 10.1007/s00018-022-04684-6.

## Introduction

Despite the numerous advances in the field of assisted reproductive technology (ART) over the last few decades, a significant number of patients still suffer from repeated implantation failure [1, 2]. Successful implantation depends on intricate crosstalk between good-quality blastocysts and the receptive endometrium that is synchronized [3]. The receptive endometrium, as a result of sophisticated molecular interactions, plays a crucial role in the embryo implantation and continuation of pregnancy, providing an optimum site on which the embryo can stably attach on time [4]. According to various ultrasonographic data or histological measurements from biopsies, endometrial thickness is positively correlated with the rates of embryo implantation and pregnancy, suggesting as a potent predictor for success in ART [5, 6]. A “thin endometrium” is mostly defined as an endometrial thickness < 7 mm and has been well recognized to be closely associated with implantation failure [7, 8]. It is mainly characterized by high uterine blood flow impedance, poor vascular endothelial growth factor (VEGF) expression, poor angiogenesis, and inadequate endometrial growth [9, 10]. Many clinical trials have been applied various adjuvant therapies, including estradiol, granulocyte colony stimulating factor (G-CSF), and aspirin, to the patients with thin endometrium [11–13]. Furthermore, recent studies have reported new approaches, such as regenerative medicine using mesenchymal stem cells (MSCs) or platelet-rich plasma (PRP), to recover the functional endometrium to the level of healthy women [10, 14]. However, there are no completely successful treatments that showed a remarkable therapeutic effect on endometrial growth and recovery leading to significant improvements in the rates of embryo implantation and pregnancy.

During implantation, growth factors play roles in temporal and spatial coordinated activation of the functional blastocysts and the receptive endometrium by binding to their specific cell surface receptors and subsequently initiating the downstream cascades [15, 16]. Among these growth factors, insulin-like growth factors (IGFs) are key components in various reproductive events in multiple mammalian species including humans, acting as mediators of steroid hormones in a paracrine and/or autocrine manner within the regulation of specific IGF binding proteins (IGFBPs) [17]. IGFBP3 is well known as a member of the IGFBP family that highly expresses in the endometrium and at the maternal–fetal interface [18]. In mice, IGFBP3 is involved in the regulation of endometrial cell proliferation and secreted by the endometrial cells to promote the embryo development by mediating the action of IGFs [19, 20]. Moreover, IGFBP3 is found its abundant expression in human endometrium during the secretory phase, especially for the window of implantation [21], suggesting its roles in embryo implantation and decidualization.

Botulinum toxin A (BoTA) is widely used in the clinics for various purposes, including plastic and aesthetic surgery, and for chronic pain relief [22–24]. Moreover, it has been recently been reported that BoTA has a positive effect on the re-epithelialization of human keratinocytes and angiogenesis of endothelial cells [25]. We have previously reported that intrauterine administration of BoTA improves the endometrial vascular reconstruction and increases the rates of embryo implantation in normal mice [26]. However, there are still no further reports evaluating the therapeutic effect of BoTA in the reconstruction of the functional layer in the thin endometrium. Lack of the functional layer of the endometrium, a crucial feature of a thin endometrium, is a well-known critical etiology leading to recurrent implantation failure and low successful pregnancy [10]. This led us to explore the therapeutic impact of BoTA administration on the pathophysiological aspects of the thin endometrium and its underlying molecular mechanisms of the beneficial effects of BoTA in recovering the structure and functionality of the thin endometrium. In this study, we developed a murine model with thin endometrium that is structurally and functionally compromised resulting in significantly delayed and suppressed fertility ability, and used this model to investigate the therapeutic impact of BoTA on recovering the thin endometrium and its underlying molecular mechanisms.

## Materials and methods

### Animal uses

All experiments were conducted under a Home Office license and the Animal Act, 1986, and had a local ethical approval for care and use of laboratory animals. C57BL/6 strain mice were maintained by strict accordance with the policies of the CHA University Institutional Animal Care and Use Committee (IACUC, approval number 210055). Five to seven-week-old female and seven to ten-week-old male mice were provided by Orientbio (Gapyeong, Gyeonggi, South Korea) and maintained under standard environmental conditions of 12 h light: 12 h dark at a controlled room temperature (RT; 20–22 °C) with free access to food and water.

### Experimentally induced mouse model of thin endometrium

A 95% ethanol (EtOH)-induced thin endometrium mouse model was established using female mice (*n* = 21) synchronized in the estrous cycle stage as previously described [27]. Prior to the surgery, the cycle was defined by daily vaginal smear and proved by the appearance and shape of keratinized vaginal epithelial cells. Selected mice were anesthetized with 2,2,2-Tribromoethanol (Avertin). A small vertical incision was made into the inferior abdomen to expose the uterus. Ethanol was prepared in 40 μl of saline at a final concentration of 95% and instilled using a 31-gauge insulin syringe into one side of mouse uterine cavities and saline was infused into the other side of uteruses for the control. Uterine tissues were harvested 2 days and 12 days after EtOH administration, respectively. To evaluate reproductive performance, nine mice with thin endometrium under different treatment conditions, female mice were placed singly with the same strain male mice overnight for the natural mating after 7 days of generation of thin endometrium. The presence of a vaginal plug the following morning (day 1 of pregnancy) was used as an indicator of successful mating. After 8 days of mating, both sides of uterine tissues were obtained and the number of implantation sites were recorded.

### Human samples

Human samples, diagnosed as thin endometrium or normal after hysteroscopy, were obtained (*n* = 4) from the CHA fertility center Bundang to validate the successful establishment of a murine model of thin endometrium via 95% EtOH intrauterine administration. Informed consent was obtained from each patient and this study was allowed by the Institutional Review Board (IRB approval number, 2020–10-007) of the CHA Bundang Medical Center.

### Intrauterine administration of BoTA and insulin-like growth factor binding protein-3

For assessment of BoTA or insulin-like growth factor binding protein-3 (IGFBP3) effects in thin endometrium, BoTA (0.5 IU or 2 IU; Botulax; Hugel, Seoul, Korea) or recombinant IGFBP3 mouse recombinant protein (4 μg, R and D system, 773-b3-025) was prepared in 40 μl of saline and infused into one side of EtOH-induced thin endometrium and saline was infused into the other side of horns for the control (n of mice for BoTA administration = 20, n of mice for IGFBP3 administration = 3). Administration of BoTA was conducted at 4 days after the induction of thin endometrium with EtOH administration. Uteruses were obtained 7 days after surgery for experiments for further analyses.

### Mouse embryo collection and co-culture with endometrial epithelial cells

For the embryo collection, C57BL/6 five female mice at 5–7 weeks old were super-ovulated by intraperitoneal injection of Pregnant Mare Serum Gonadotropin (PMSG 10 IU, DAESUNG Microbiological, Korea), followed by Human Chorionic Gonadotropin (hCG 5 IU, Sigma) 44–46 h later. Mice were placed overnight singly with fertile males. Fertilized one-cell embryos were obtained by tearing the ampulla and releasing them into 0.1% hyaluronidase and M2 solution (Sigma, USA) to wash cumulus cells. Collected embryos were cultured in a 20 μl drop of KSOM (Millipore, USA) covered with mineral oil at 5% CO_2_, 37 °C until the blastocyst stage. All embryos were manipulated using a sterile Pasteur pipette. Only fully expanded blastocysts with clearly observable inner cell mass and trophectoderm on day 5 were included for further analyses. To evaluate the stability of attached embryo depending on treatment conditions, fully expanded blastocysts were co-cultured with confluent Ishikawa cells, which were plated on 1% matrigel-coated cover glass, and the stability of attached embryo was assessed using a scoring scale at indicated time points including 19, 24, 42, and 48 h [26, 28, 29].

### Cell culture and EtOH- or BoTA treatment

Human endometrial epithelial cells (Ishikawa) purchased from American Type Culture Collection (ATCC) were maintained as previously described [29]. The optimum percentage and time of EtOH application was validated by a screening test using Ishikawa cells. Cells were treated with EtOH at a final concentration of 10% for up to 2 min to mimic the in vivo uterine environment of a thin endometrium established by 95% EtOH intrauterine administration. For the evaluation of BoTA effects, cells were treated 2 IU BoTA.

### Immunohistochemistry and microscopy

Histology and immunohistochemistry were performed as previously described [26]. In brief, paraffin-embedded tissue Sects. (5 μm) were deparaffinized in Histoclear for 10 min and rehydrated through a descending gradient of ethanol. After washing, sections were then delimited with a DAKO pen. Endogenous peroxidase activity was blocked using 3% H_2_O_2_ in methanol for 10 min at room temperature. Antigen retrieval was performed in sodium citrate buffer (pH 6) for 25 min in the microwave. Subsequently, non-specific binding sites were blocked with 5% bovine serum albumin (BSA) in PBS for 1 h. Sections were incubated with primary antibody against COL1A1 (Santacruz, sc-293182, 1:200), Ki67 (BD Pharmingen, 550609, 1:100), IGFBP3 (Novus; NBP2-12,364, 1:200), OPN (Enzo; ADI-905–629, 1:100), and active-OPN (24H5L3, Invitrogen; 702184, 1:200) overnight at 4 °C; the samples were bound with secondary antibodies (Vector laboratories; ZG0122, 1:200) for 1 h at room temperature. The signal was detected by DAB substrate solution (Vector laboratories; SK-4100, DAB substrate kit). Sections were briefly counter-stained with hematoxylin and mounted. Images were acquired and measured under a biological microscope (Olympus CKX-53).

### Masson’s trichrome staining

To measure the levels of fibrosis induced after 95% EtOH infusion, staining was conducted using Masson’s trichrome staining kit (DAKO; AR173) according to the manufacturer’s protocol. Deparaffinized tissue sections were stained with Wiegert’s hematoxylin for 5 min, then smooth muscle with Biebrich Scarlet-acid fuchsin for 10 min and immersed in phosphomolybdic acid for 15 min at 40 °C. Collagen was stained with Aniline blue for 10 min at 35 °C.

### Immunofluorescence

Deparaffinized tissue samples were rehydrated through graded ethanol. Antigen retrieval was performed in sodium citrate buffer (pH 6) for 25 min in the microwave. After washing, samples were then delimited with a DAKO pen. Subsequently, non-specific binding sites were blocked with 5% bovine serum albumin (BSA) in PBS for 1 h. Sections were incubated overnight at 4 ℃ using primary antibodies against CD31 (Abcam; 28364, 1:100) and Ki67 (BD Biosciences; 550609, 1:1000). Goat anti-rabbit or anti-mouse secondary antibodies were applied for 1 h at room temperature in dark, and then sections were stained with DAPI (Thermo; 62248, 1:1000) for 10 min and mounted with a mounting medium (DAKO; S3025). Tissue sections were imaged using a confocal microscope (Carl Zeiss; Oberkochen, Germany) and tile scans were performed when required. Images were analyzed using ZEN black edition software (Carl Zeiss).

### Quantitative RT-PCR-based analysis of mRNA expression

Uterine tissues were homogenized in 600 μl of TRIzol reagent (Ambion; Life Technologies Corporation, CA, USA) and total RNA at 1 μg was converted to complementary DNA using Superscript IV (Invitrogen; 18091050). Amplifications were performed in a CFX Connect Real-time PCR Detection System (Bio-Rad; Hercules, CA, USA) using SYBR Green (Roche; Basel, Switzerland). All reactions were conducted in triplicate, and the relative mRNA expression level was normalized to housekeeping gene *Actb* or *Rpl7* mRNA expression. Primer sequence pairs used for these analyses are shown in Table S1.

### Immunoblotting analysis

Immunoblotting analyses were conducted as previously described [29]. In brief, uterus tissues were minced by a homogenizer, lysated with ice-cold RIPA buffer, and protein lysates were separated by SDS-PAGE and transferred onto PVDF membrane (Millipore; Billerica, MA.). After blocking in 5% BSA in 1X TBS buffer containing 0.1% Tween-20 for 1 h at RT, the membranes were incubated with primary antibodies against integrin β3 (Cell Signaling; #13166, 1:1000), OPN (Enzo; ADI-905–629, 1:1000), VEGF (Invitrogen; MA5-13182, 1:500), IGFBP3 (Novus; NBP2-12364, 1:500), and β-actin (Cell signaling; 3700S, 1:1000) for overnight, washed and reacted with HRP-conjugated mouse or rabbit secondary antibodies (BIORAD; 170–6516, 170–6515, 1:3000). Signals were developed using ECL solution (Thermo; 32106) and immunoreactive bands were visualized by LAS-4000. Quantification of the intensity of bands compared to the loading control was conducted by using Image J.

### Library preparation of EtOH-treated uterus and sequencing for RNA-seq analysis

For control (saline-treated mouse uterus tissues, *n* = 3) and test (EtOH-treated mouse uterus tissues, *n* = 3) RNAs, the construction of library was performed using QuantSeq 3’ mRNA-Seq Library Prep Kit (Lexogen, Inc., Austria) according to the manufacturer’s instructions. Each 500 ng total RNA was extracted from 3 different sets of saline-treated and EtOH-treated uterine tissues and an oligo-dT primer containing an Illumina-compatible sequence at its 5′ end was hybridized to the RNA and reverse transcription was performed. After degradation of the RNA template, second strand synthesis was initiated by a random primer containing an Illumina-compatible linker sequence at its 5’ end. The double-stranded library was purified by using magnetic beads to remove all reaction components. The library was amplified to add the complete adapter sequences required for cluster generation. The finished library is purified from PCR components. High-throughput sequencing was performed as single-end 75 sequencing using NextSeq 500 (Illumina, Inc., USA). The raw and normalized data have been deposited in the Gene Expression Omnibus (GEO) data base (accession number: GSE 207379).

### Data analysis

QuantSeq 3′ mRNA-Seq reads were aligned using Bowtie2 [30]. Bowtie2 indices were either generated from genome assembly sequence or the representative transcript sequences for aligning to the genome and transcriptome. The alignment file was used for assembling transcripts, estimating their abundances and detecting differential expression of genes. Differentially expressed gene were determined based on counts from unique and multiple alignments using coverage in Bedtools [31]. The RC (Read Count) data were processed based on quantile normalization method using EdgeR within R using Bioconductor [32]. Gene classification for Gene Ontology (GO) and pathway analysis was performed by DAVID (http://david.abcc.ncifcrf.gov/) and Medline databases (http://www.ncbi.nlm.nih.gov/). Classified genes and their fold enrichment values were plotted with ggplot2 packages in R (version 3.42) and automatically categorized by ClueGO [33, 34]. Moreover, gene–gene interaction was visualized by Gene mania databases (http://genemania.org/data/) [35]. The significance cutoffs were set for fold-change (≥ 2.0), *P* value (< 0.05) and FDR (< 0.05).

### Karyotyping analysis

To ascertain the safety of the BoTA intrauterine administration, a karyotype analysis was conducted by the Animal Resources Research Center of Konkuk University of South Korea. For sample preparation, BoTA was administered into both uterine horns of 5–7 weeks old C57BL/6 females (*n* = 6). After 1 week of infusion, mice were mated. On gestational day 13, the pregnant uteruses were harvested to obtain mouse embryonic fibroblasts (MEFs). Uterine decidua was removed and separated from each embryo. Then heads, legs, and hands were decapitated from the embryonic bodies and internal organs were removed. Minced embryonic bodies were mixed with TryPLE by gently pipetting up and down for dissociation and incubated for 5 min at 37 °C. After washes with fresh PBS, single cells were collected and cultured in DMEM/F12 (Gibco; Grand Island, NY, USA) containing 20% heat-inactivated fetal bovine serum and 1% penicillin–streptomycin (Gibco; Grand Island, NY, USA), 1% L-glutamine at 37 °C, 5% CO_2_. At 95% confluency, cells were treated with Colcemid (Gibco, 10 μg/ml) for 3 ~ 5 h to arrest at metaphase. Subsequently, hypotonic solution (0.075 M KCL + 1% sodium citrate) was treated at 37 ℃ for 25 min. Harvested cells were centrifuged at 1500 rpm for 10 min, supernatant was discarded, and fixative (Methanol: Acetic acid = 3:1) was added. Washing with fresh fixative was performed more than twice. After last change of fixative, the cell suspension was dropped on cold wet slide. The slides were air-dried and treated with trypsin. Giemsa (GTG-banding method) staining was conducted. Each chromosome was observed under light microscope.

### Statistical analysis

Data were analyzed using the GraphPad Prism 9.0 software (La Jolla, CA, USA). Comparison groups were analyzed with unpaired Student *t*-test for parametric distributions. For multiple comparisons, the ordinary one-way ANOVA analysis with Dunnett’s multiple comparison test or two-way ANOVA analysis with Tukey’s multiple comparisons test. For all cases, a *P* value that was < 0.05 was considered statistically significant (*P* < 0.05 (*), *P* < 0.01 (**), *P* < 0.001 (***) and *P* < 0.0001 (****)).

## Results

### Establishment of an EtOH-induced thin endometrium murine model

**Fig. 1:**
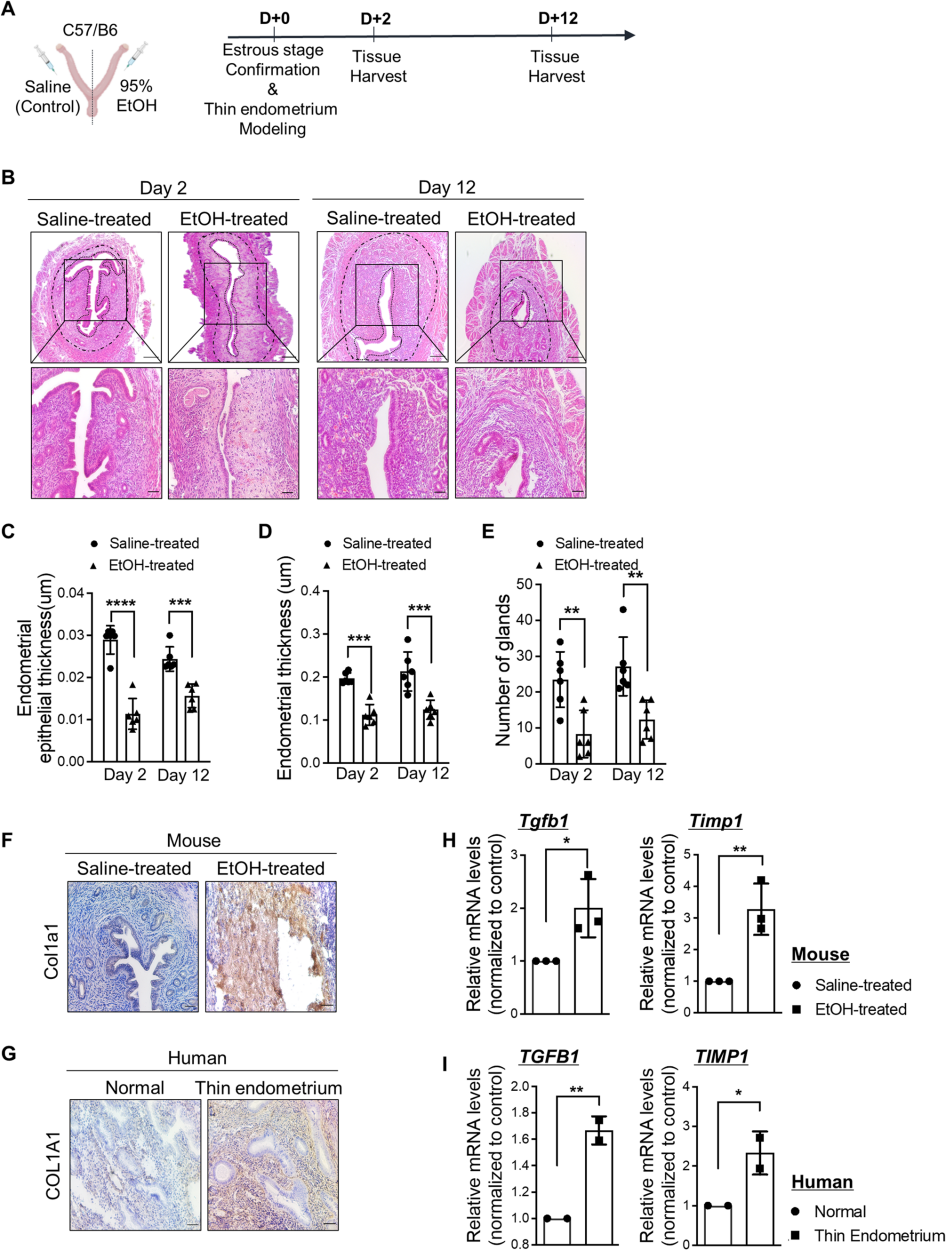
Establishment of an EtOH-induced thin endometrium murine model (**A**) An experimental timeline of thin endometrium generation in mice (**B**) H&E-stained images following treatments with saline (control) and 95%-EtOH (thin endometrium) on Day 2 and 12. Upper panel shows the entire region of each uterus and magnified images (black square) are shown in lower panel. Dash-dotted lines indicate the whole endometrium and dotted lines indicate the lining of the endometrial epithelium. Scale bar: 100, 200 μm. Graphs displaying quantified data of average endometrial epithelial thickness (**C**), endometrial thickness (**D**), and total number of glands (**E**) of EtOH-treated endometrium compared to saline-treated control. Data shown for (**C**–**E**) are from 3 independent experiments (3 independent EtOH-treated uteri from 2 independent mice), presented as mean ± SD, and analyzed by two-way ANOVA with Tukey’s multiple comparisons test including *P* values (* < 0.05, ** < 0.01, *** < 0.001, **** < 0.0001). Immunostaining of Col1a1 (COL1A1) in endometrial tissues obtained from mouse (saline-treated vs. EtOH-treated) (**F**) and human (normal (*n* = 1) vs. thin endometrium (*n* = 1)) (**G**), Scale bar; 100 μm. QRT-PCR analyses of *Tgfb1* (*TGFB1*) and *Timp1* (*TIMP1*) in mouse (saline-treated vs. EtOH-treated endometrium) (**H**) and human (normal (*n* = 2) vs. thin endometrium (*n* = 2)) (**I**)

We established an experimentally induced murine model with thin endometrium via intrauterine administration of 95% ethanol (EtOH), which was adopted and modified from previous reports [27, 36, 37]. For the validation of successful modeling, 95% EtOH was infused into one side of the mouse uterine horns and saline was infused into the other side of the horns for the control in mice, which were in the estrous cycle. Mouse uterine tissues were obtained 2 or 12 days after modeling of the thin endometrium (Fig. 1A). To evaluate the pathophysiological features of the 95% EtOH-induced thin endometrium compared to saline-treated control, the thickness of the whole endometrium and endometrial epithelium, and the total number of glands were assessed. Histological evaluation revealed that endometrial epithelial thickness (Day 2; 0.48-fold down; *P* = *0.0001*, Day 12; 0.62-fold down; *P* = *0.0004*), endometrial thickness (Day 2; 0.57-fold down; *P* = *0.0002*, Day 12; 0.58-fold down; *P* = *0.0004*), and number of glands (Day 2; 0.37-fold down; *P* = *0.00049*, Day 12; 0.42-fold down; *P* = *0.00017*) were significantly reduced in the 95% EtOH-treated endometrium compared to the saline-treated group (control) both on days 2 and 12 of EtOH administration into the uterine cavity (Fig. 1B–E and Supplemental Fig. S1A). The EtOH-treated uterus displayed uncertain boundaries of epithelial, stromal, and myoma layers with dilated and irregular gland shapes, whereas the control group exhibited the clear three distinct layers with tubular-shaped glands (Fig. 1B). We set the experimental design to evaluate the recovery period of approximately 3-estrous cycles (12 days) from the time of modeling in mice with EtOH-induced thin endometrium. Interestingly, the thickness of the whole endometrium and endometrial epithelium remained similar even after a period of three estrous cycles in both the control and EtOH-treated endometrium (Fig. 1B–D), implying that reduced endometrial thickness mediated by EtOH administration remained unaffected by endometrial dynamic changes during the estrous cycle. Moreover, fibrotic lesions were observed with accumulated collagen in the stromal layer (Fig. 1F–G), and higher expression levels of both transforming growth factor β-1 (*Tgfb1*) and TIMP metallopeptidase inhibitor 1 (*Timp1*) were detected in EtOH-treated endometria compared to saline-treated controls, which were key features observed in patients with thin endometria (Fig. 1H–I and Supplemental Fig. S1B).

### Decreased embryo implantation rates with significantly reduced endometrial receptivity and angiogenesis in the EtOH-induced thin endometrium in mice

**Fig. 2:**
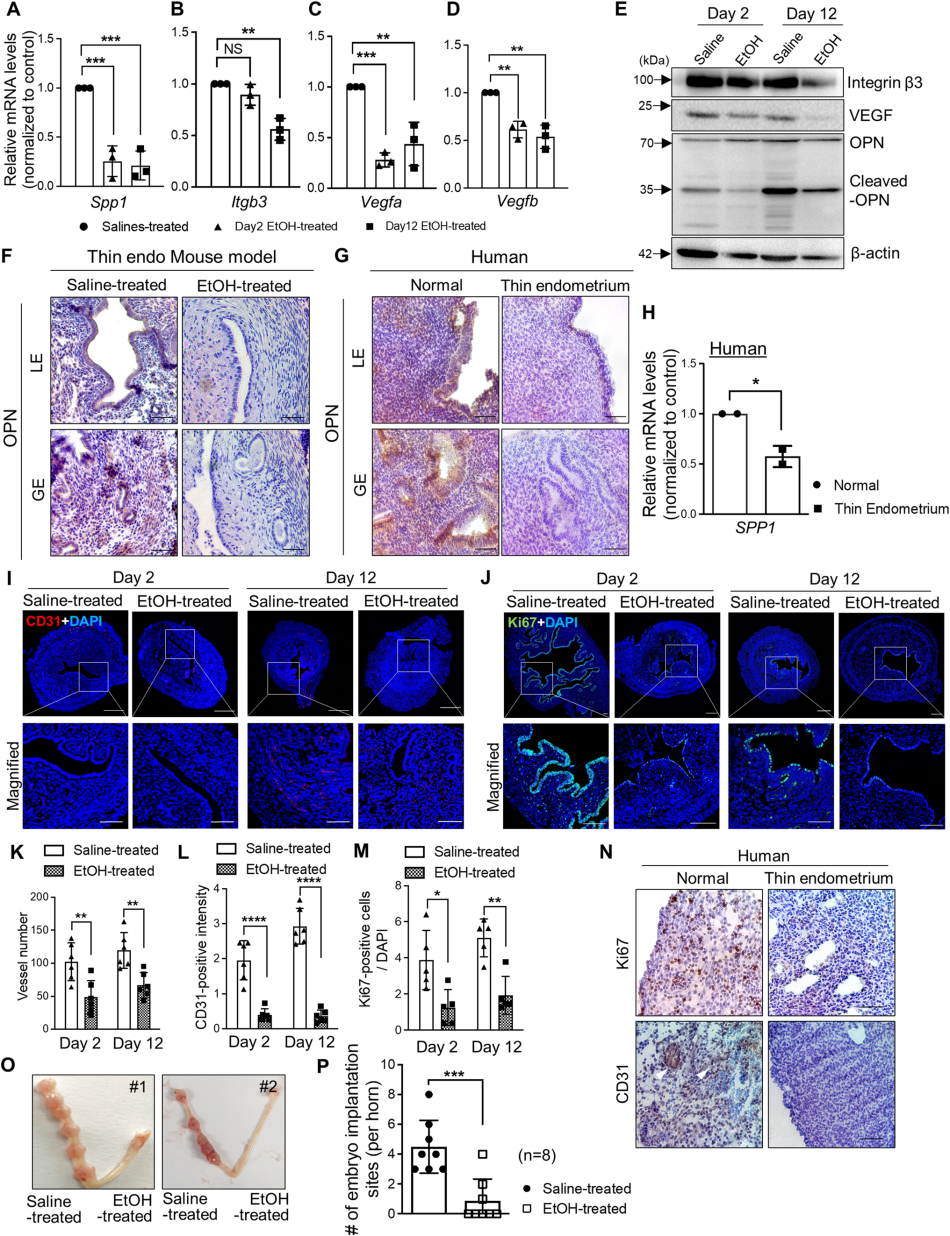
Decreased embryo implantation rates with significantly reduced endometrial receptivity and angiogenesis in EtOH-induced thin endometrium in mice (**A**–**D**) QRT-PCR analyses of *Spp1*, *Itgb3*, *Vegfa*, and *Vegfb* in EtOH-treated groups (Day 2 and Day 12) compared to saline-treated control. Data are presented as mean ± SD and analyzed using the ordinary one-way ANOVA with Dunnett’s multiple comparisons test including *P* values (* < 0.05, ** < 0.01, *** < 0.001, **** < 0.0001). **E** Immunoblotting assays for Integrin β3, VEGF, and OPN in EtOH-treated endometrium compared to saline-treated group. β-actin was used as a loading control. Immunohistochemical (IHC) images of OPN in the regions of luminal (LE) and glandular epithelium (GE) from the mouse endometrial tissues (saline-treated vs. EtOH-treated) (**F**) and human samples (normal vs. thin endometrium) (**G**). Scale bar; 100 μm. **H** QRT-PCR analysis of *SPP1* in human thin endometrium compared to normal endometrium (*n* = 2). Immunofluorescence (IF) staining for CD31 (**I**; Red) and Ki67 (**J**; Green) in EtOH-treated endometrium compared to saline-treated control on Day 2 and 12. Upper panel shows the entire region of each uterus and magnified images are shown in lower panel. Nuclei staining (Blue). Scale bar: 100 μm (upper panel), 200 μm (lower panel). **K** Quantification of total vessel number in the cross-sectional endometrial tissues in saline-treated vs. EtOH-treated endometrium (Day 2, Day 12). CD31-positive intensity (**L**) and Ki67-positive cells (**M**) are normalized to DAPI nuclei expression and quantified. Data shown in (**K**–**M**) are presented as mean ± SD and analyzed by two-way ANOVA with Tukey’s multiple comparisons test including *P *values (* < 0.05, ** < 0.01, *** < 0.001, **** < 0.0001). **N** IHC analyses of Ki67 in human thin endometrium compared to normal tissues. Scale bar; 100 μm. **O** Representative images of uteri with implantation sites on pregnancy day 8 with EtOH-administration compared to saline-treatment. The number of implantation sites (per uterine horn) on pregnancy day 8 are quantified in a graph (**P**). Data are analyzed by unpaired *t*-test including *P* values (* < 0.05, ** < 0.01, *** < 0.001, **** < 0.0001)

Adequate endometrial growth is a critical factor for successful embryo implantation and maintenance of pregnancy. It is generally accepted that successful pregnancy does not usually occur when the endometrial thickness is < 7 mm [38]. Additionally, endometrial thickness < 6 mm is associated with a decreased probability of achieving a full-term pregnancy [39]. However, evidence for molecular biological characteristics of thin endometrium other than clinical features is still insufficient. Thin endometrium induced by EtOH administration into the uterine cavity in mice demonstrated that reduced expression of endometrial receptivity or angiogenesis-related genes including OPN (*Spp1*), integrin β3 (*Itgb3*), and VEGF (*Vegfa* and *Vegfb*) at both the mRNA and protein levels (Fig. 2A–E, and Supplemental Fig. S2A–D). In particular, the level of OPN (*Spp1*) was significantly decreased in the endometrial tissues two days after the induction of thin endometrium and this pattern was lasted until day 12. Moreover, the expression level of the cleaved fragment of OPN (35 kDa) was dramatically reduced in the EtOH-induced thin endometrium (Fig. 2E). Lower levels of OPN in both the luminal (LE) and glandular epithelium (GE) were detected in EtOH-treated endometrium compared to the saline-treated control, which was consistently observed in human thin endometrial tissues compared to normal tissues (Fig. 2F–H). Endometrial angiogenesis and cellular proliferation were also reduced in the EtOH-treated group (Fig. 2I–J). Descending angiogenic and proliferative effects were further quantified by counting the total number of vessels, CD31-positive areas, and Ki67-positive cells (Fig. 2K–M). Likewise, a dramatically reduced numbers of proliferative cells and CD31-positive cells were observed in patient with thin endometrium (Fig. 2N). Moreover, a significantly lower number of embryo implantation sites (*P* < *0.0001*; n of mice = 12, n of implantation sites = 65) was observed in the EtOH-induced thin endometrium than in the saline-treated endometrium in mice (Fig. 2O–P).

### Identification of alterations in gene expression detected in EtOH-induced thin endometrium

**Fig. 3:**
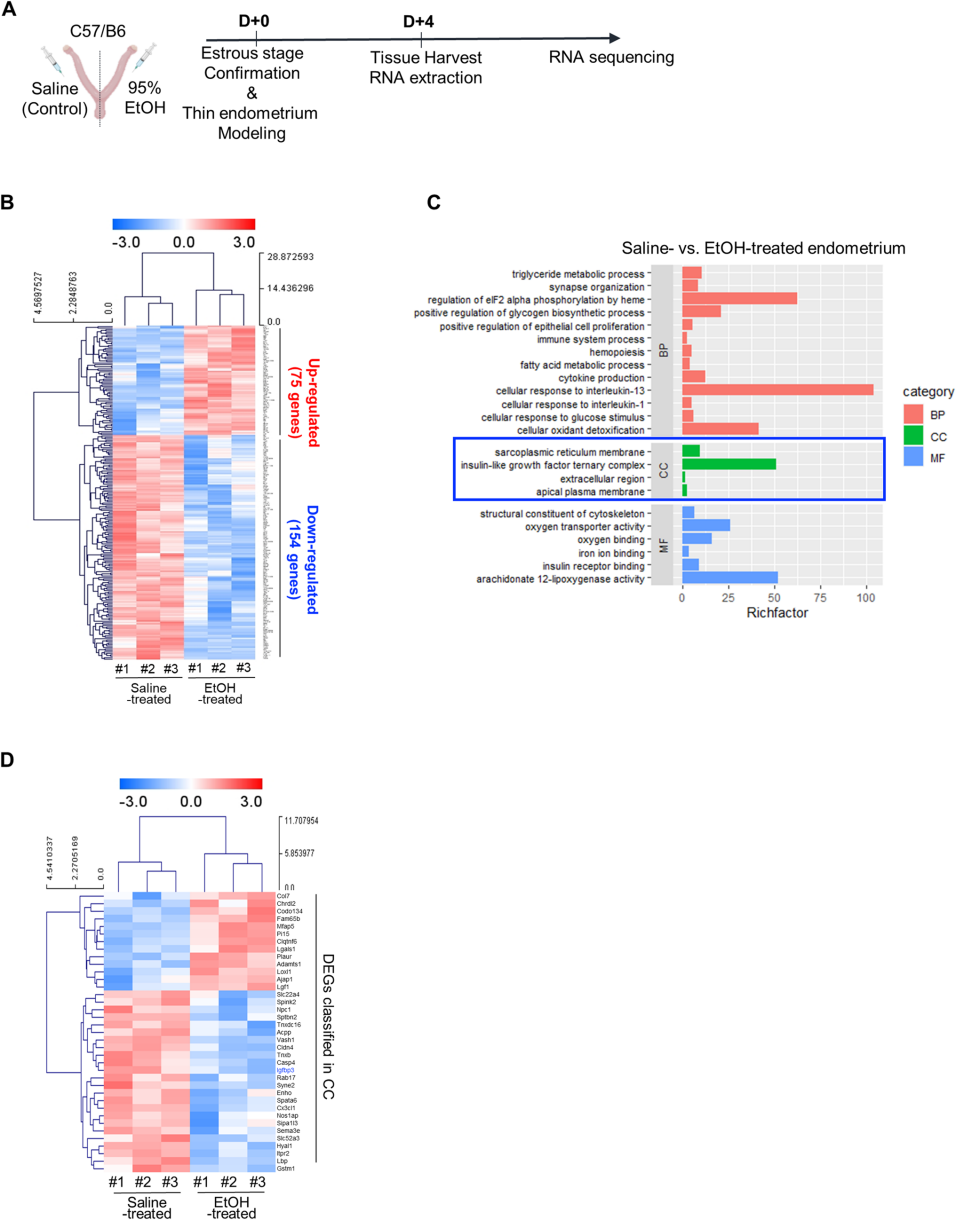
Identification of alterations in gene expression detected in EtOH-induced thin endometrium **A** An experimental schedule for RNA sequencing. **B** Heatmap of 229 differentially expressed genes in EtOH-treated uteri compared to saline-treated uteri. Each column represents a distinct sample (3 samples from saline-treated uteri, EtOH-treated uteri) and each row represents an individual gene. **C** Plot displaying biological process (BP), cellular component (CC), molecular function (MF) of gene ontology (GO) and pathway analysis of differentially expressed genes of EtOH-treated uteri compared to saline-treated groups using DAVID tool and their fold enrichment on *X*-axis. **D** Heatmap of DEGs from cellular component (CC) terms shown in Fig. 3C

To further examine the alterations in global gene expression depending on the endometrial thickness in EtOH-induced thin endometrium compared to saline-treated control, RNA-seq analysis data were generated from the EtOH-treated versus saline-treated endometrial tissues obtained from three independent uteri; each pair was harvested from one mouse (Fig. 3A). Unsupervised hierarchical clustering analyses using a fold change cutoff of 2 and a *P*-value cutoff of 0.05 identified a total of 229 genes (75 genes were up-regulated (red) and 154 genes were down-regulated (blue)) in EtOH-treated thin endometrium compared to control (Fig. 3B and Supplementary Table S2). Functional clustering of 229 differentially expressed genes (DEGs) using the Database for Annotation, Visualization and Integrated Discovery (DAVID) online tools demonstrated the enriched gene ontology (GO) and pathways in the EtOH-induced thin endometrium compared with the control. Enriched GO terms in each category and pathway including associated-gene counts, *P* values, and fold enrichment (FE), are shown in Table 1. The *P* values and FE-values were calculated using Fisher’s exact test and multiple comparisons test, respectively (*P* < 0.05 and FE > 1.5). A total of 229 DEGs were classified and annotated according to the GO terms of biological process (BP), cellular components (CC), and molecular functions (MF) (Fig. 3C and Table 1). Specific BP categories demonstrate that metabolic processes including triglyceride metabolism (GO:0006641, *P* = *0.0061*), glycogen biosynthesis (GO:0045725, *P* = *0.0088*), fatty acid metabolism (GO:0006631, *P* = *0.017*), and cellular response to glucose stimulus (GO:0071333, *P* = *0.031*), and immune system processes (GO:0002376, *P* = *0.031*), cytokine production (GO:0001816, *P* = *0.024*), cellular response to interleukin-13 (GO:0035963, *P* = *0.019*) and 1 (GO:0071347, *P* = *0.041*) involved in immune responses were enriched in tissues obtained from the EtOH-induced thin endometrium compared to the saline-treated group. Moreover, alterations of the genes involved in the pathways of synapse organization (GO:0050808, *P* = *0.049*) and positive regulation of epithelial cell proliferation (GO:0050679, *P* = *0.036*) were significantly abundant in the EtOH-induced thin endometrium, displaying an aberrance in the endometrial cellular integrity and proliferative capacity in thin endometrium. The enriched terms in the CC category included integral components of the extracellular region (GO:0005576, *P* = *0.035*), apical plasma membrane (GO:0016324, *P* = *0.042*), and insulin-like growth factor ternary complex (GO:0042567, *P* = *0.038*). MF terms were composed of the oxygen transporter activity (GO:0005344, *P* = *0.00044*), structural constituent of the cytoskeleton (GO:0005200, *P* = *0.0067*), insulin receptor binding (GO:0005158, *P* = *0.044*), and arachidonate 12-lipoxygenase activity (GO:0106237, *P* = *0.038*) (accession number: GSE207379). These analyses implicate that aberrant endometrial metabolism and immune responses accompanied by structural impairment might be major pathophysiological features consistent with the previous reports on human thin endometrium [10, 40, 41]. Interestingly, our interrogation on RNA-seq analyses revealed that the enriched CC components, including the apical plasma membrane, extracellular region, and insulin-like growth factor ternary complex identified in EtOH-induced thin endometrium, were shared with DEGs identified from BoTA-treated endometrial tissues compared to the control, which we previously reported (accession number: GSE146934) [26] (Fig. 3C–D, Supplemental Fig. S3A, B, and Tables S3, 4). This promptly led us to examine whether BoTA administration had a beneficial effect on uterine structural and functional repair in an experimentally-induced mouse model with thin endometrium.

**Table 1:** Whole Gene ontology (GO) and pathway analysis of differentially expressed genes of EtOH-treated endometrial samples compared to control using DAVID tool

GO Category	Term	Count	*P* value	Fold enrichment (FE)
Biological process (BP)	GO:0006641 ~ triglyceride metabolic process	4	6.10E^−03^	10.7
Biological process (BP)	GO:0050808 ~ synapse organization	3	4.90E^−02^	8.4
Biological process (BP)	GO:0010999 ~ regulation of eIF2 alpha phosphorylation by heme	3	8.90E^−04^	62.4
Biological process (BP)	GO:0045725 ~ positive regulation of glycogen biosynthetic process	3	8.80E^−03^	20.8
Biological process (BP)	GO:0006631 ~ fatty acid metabolic process	6	1.70E^−02^	4
Biological process (BP)	GO:0050679 ~ positive regulation of epithelial cell proliferation	4	3.60E^−02^	5.5
Biological process (BP)	GO:0030097 ~ hemopoiesis	4	4.90E^−02^	4.8
Biological process (BP)	GO:0071347 ~ cellular response to interleukin-1	4	4.10E^−02^	5.2
Biological process (BP)	GO:0098869 ~ cellular oxidant detoxification	2	4.70E^−02^	41.6
Biological process (BP)	GO:0035963 ~ cellular response to interleukin-13	2	1.90E^−02^	103.9
Biological process (BP)	GO:0001816 ~ cytokine production	3	2.40E^−02^	12.5
Biological process (BP)	GO:0071333 ~ cellular response to glucose stimulus	4	3.10E^−02^	5.9
Biological process (BP)	GO:0002376 ~ immune system process	9	3.10E^−02^	2.4
Cellular component (CC)	GO:0005576 ~ extracellular region	26	3.50E^−02^	1.5
Cellular component (CC)	GO:0,042,567 ~ insulin-like growth factor ternary complex	2	3.80E^−02^	50.9
Cellular component (CC)	GO:0033017 ~ sarcoplasmic reticulum membrane	3	4.10E^−02^	9.3
Cellular component (CC)	GO:0016324 ~ apical plasma membrane	8	4.20E^−02^	2.5
Molecular function (MF)	GO:0005344 ~ oxygen transporter activity	4	4.40E^−04^	26
Molecular function (MF)	GO:0019825 ~ oxygen binding	4	1.90E^−03^	16
Molecular function (MF)	GO:0005200 ~ structural constituent of cytoskeleton	5	6.70E^−03^	6.7
Molecular function (MF)	GO:0005506 ~ iron ion binding	7	1.60E^−02^	3.4
Molecular function (MF)	GO:0106237 ~ arachidonate 12-lipoxygenase activity	2	3.80E^−02^	51.9
Molecular function (MF)	GO:0005158 ~ insulin receptor binding	3	4.40E^−02^	8.9

The cutoff for significance was set by *P* value < 0.05 and FE fold enrichment  > 1.5, *BP* biological process, *CC* cellular component, and *MF* molecular function annotations

### Recovery of reduced fertility rates in the thin endometrium via intrauterine BoTA administration

**Fig. 4:**
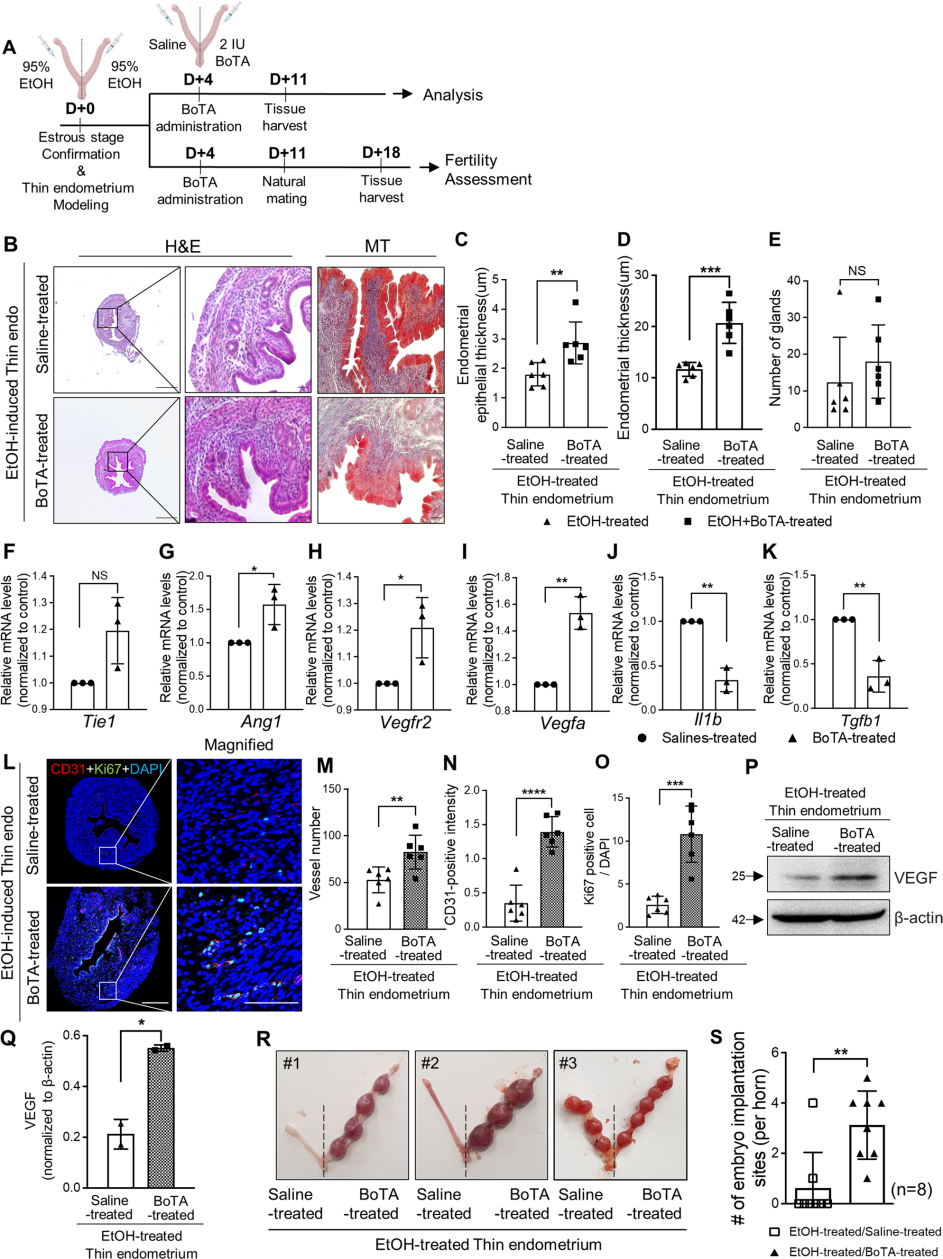
Recovery of reduced fertility rates in the thin endometrium via intrauterine BoTA administration **A** An experimental schedule for the evaluation of the effects of intrauterine BoTA administration. **B** H&E and MT staining of BoTA-treated endometrium compared to saline-treated one in EtOH-induced thin endometrium. Quantification of histological evaluations of endometrial epithelial thickness (**C**), endometrial thickness (**D**), and total number of glands (**E**) in saline-treated vs. BoTA-treated thin endometrium. (**F-K**) QRT-PCR analyses of angiogenesis and anti-inflammatory related markers including *Tie1*, *Ang1*, *Vegfr2*, *Vegfa*, *Il1b*, and *Tgfb1* in BoTA-treated thin endometrium compared to saline-treated group. **L** Co-immunofluorescence staining of CD31 (red) and Ki67 (green) in BoTA-treated EtOH-induced thin endometrium compared saline-treated groups. Images of white box are magnified in the right panel. Scale bar: 100 µm. Total number of vessels (**M**), CD31-positive intensity (**N**), Ki67-positive cells (**O**) are quantified. **P** Immunoblot analysis of VEGF in BoTA-treated thin endometrium compared to saline-treated thin endometrium. Densitometry of VEGF expression normalized to β-actin is shown in a graph (**Q**). **R** Representative images of uteri with implantation sites on pregnancy day 8 in mice with EtOH-treated thin endometrium with saline or BoTA treatment. The number of implantation sites is quantified in a graph (**S**). Data are analyzed by unpaired *t-*test including *P* values (*< 0.05, **< 0.01, ***< 0.001, ****< 0.0001, NS; not significant)

To examine the endometrial regenerative therapeutic effects of BoTA in thin endometrium, BoTA (2 IU) was administered into one side of the uterine horns four days after the experimental induction of thin endometrium with EtOH administration in mice, and saline was infused into the other side horn for the control. After another one week, both sides of the uterine tissues were harvested for further histological and molecular evaluations (Fig. 4A). Remarkably, the thickness of the whole endometrium and endometrial epithelial region was significantly increased with BoTA administration, however, no significant differences in the number of glands were observed between BoTA- and saline-treated uteri (Fig. 4B–E). Moreover, fibrotic lesions in the EtOH-induced thin endometrium were noticeably reduced by BoTA treatment (Fig. 4B). Intriguingly, administration of a lower concentration of BoTA (0.5 IU) failed to induce the recovery of endometrial thickness in EtOH-induced thin endometrium of mice (Supplemental Fig. S4A-E). Furthermore, BoTA treatment promoted the mRNA levels of angiogenic factors including *Tie1*, *Ang1*, *Vegfr2* and *Vegfa*, and alleviated pro-inflammatory related factors such as *Il1b* and *Tgfb1* (Fig. 4F–K). Co-immunofluorescence (co-IF) analyses of CD31 and Ki67 demonstrated that BoTA-administered EtOH-induced thin endometrium showed higher numbers of vessel formation (1.56-fold, *P* = 0.010) and increased cell proliferative capacity (4.16-fold, *P* = 0.002) especially in the region close to the epithelium, compared to the saline-treated uterus (Fig. 4L–O). Consistently, increased endometrial angiogenesis was validated with higher expression of VEGF in BoTA-treated thin endometrium compared to the control (Fig. 4P–Q).

Patients with a thin endometrium are often suffering from infertility, which is mainly caused by a deteriorated uterine environment resulting from weak endometrial receptivity, lower angiogenesis, and poor growth of the endometrial epithelium [9]. We previously confirmed that intrauterine administration of BoTA enhances the rates of embryo implantation [26]. This led us to investigate whether BoTA-induced recovery of the functional layer in the thin endometrium could continue to rescue the impaired embryo implantation. We first performed the embryo attachment assays to assess the effect of BoTA on fertility enhancement using an in vitro co-culture system [42]. Fifty-nine mouse blastocysts were transferred onto confluent Ishikawa cells, which were treated with BoTA for 5 h following the administration of 10% EtOH for up to 2 min to mimic the uterine environment of the EtOH-induced mouse thin endometrium. The stability of embryo attachment was scored up to 48 h (Supplemental Fig. S4F). Fully expanded and similar sized mouse blastocysts were chosen to rule out the embryonic issues with poor in vitro developmental potential. As expected, lower rates of embryo attachment stability were observed as the time period of EtOH treatment increased (Supplemental Fig. S4G), implying a negative correlation between the severity of the thin endometrium and the success rates of embryo implantation. No significant differences were detected among the three groups, i.e., saline-, EtOH-, or BoTA treatment following the exposure to EtOH up to 24 h of co-culture, however embryos transferred onto EtOH-exposed Ishikawa cells showed significantly unstable attachment between 42 and 48 h of time point. Interestingly, embryos transferred onto the BoTA- treated cells (5 h of exposure to BoTA following the exposure to EtOH prior to embryo co-culture) were more stably attached, which was similar to the levels observed in the control groups (Supplemental Fig. S4H). Of note, hatching failure of embryos was frequently observed in those transferred onto EtOH-treated Ishikawa cells, whereas it was not detected in BoTA-treated cells, suggesting that the uterine environment may affect embryonic development at the maternal–fetal interface. We next examined the therapeutic effect of BoTA administration on pregnancy outcomes in a thin endometrium mouse model. BoTA was administered into the EtOH-induced thin endometrium in the same manner as shown in Fig. 4A. After 7 days of intrauterine BoTA administration, the mice were naturally mated, and the uteri were dissected at pregnancy day 8. Remarkably, significantly reduced rates of embryo implantation sites (*P* = *0.0028;* n of mice = 8, n of implantation sites = 26) observed in the EtOH-induced thin endometrium were approximately 400% restored with intrauterine BoTA administration (Fig. 4R–S).

### BoTA-induced endometrial regeneration mediated by IGFBP3-dependent OPN proteolytic cleavage in a thin endometrium mouse model

**Fig. 5:**
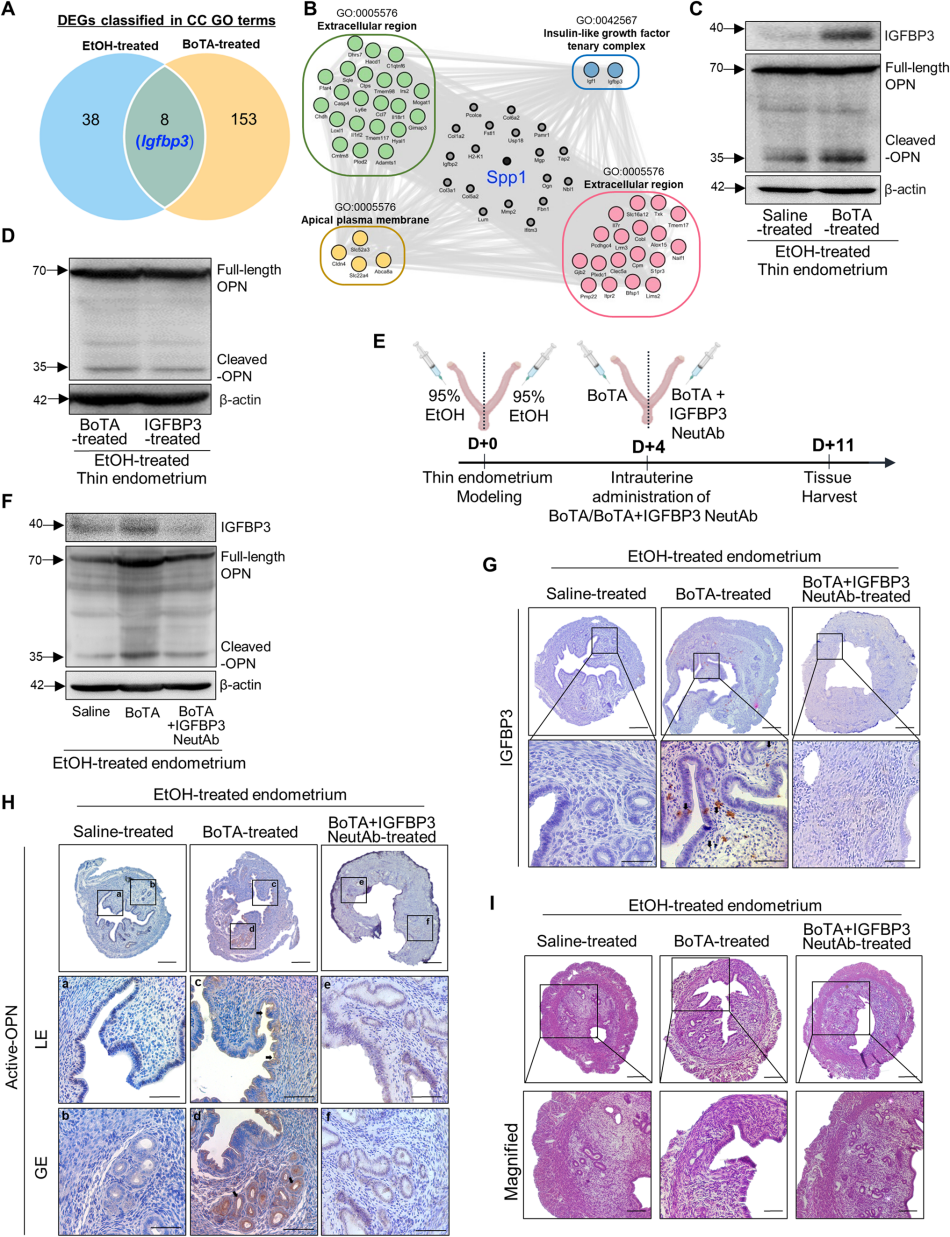
BoTA-induced endometrial regeneration mediated by IGFBP3-dependent OPN proteolytic cleavage in a thin endometrium mouse model **A** Venn diagram of DEGs from EtOH-treated vs. saline-treated uteri (GSE207379) and BoTA-treated vs. saline-treated (GSE146934) displaying a shared gene with a reciprocal expression; *Igfbp3*. **B** Gene–gene network analysis of DEGs of EtOH-treated vs. saline-treated uteri categorized in CC GO terms displaying a shared gene; *Spp1* **C** Immunoblotting analysis of IGFBP3 and OPN in BoTA-treated thin endometrium compared to saline-treated group. **D** Immunoblotting analysis of OPN in BoTA-treated thin endometrium compared to IGFBP3-treated thin endometrium. β-actin was used as a loading control. **E** An experimental schedule for an application of IGFBP3 neutralizing antibody together with BoTA. **F** Immunoblotting analysis of IGFBP3 and OPN in response to IGFBP3 neutralizing antibody treatment. β-actin was used as a loading control. **G** Immunostaining of IGFBP3 in saline-treated, BoTA-treated, and BoTA with IGFBP-3 neutralizing antibody-treated thin endometrium. Upper panel shows the expression of IGFBP3 in the whole region of uterus and the indicated region with a box is magnified in lower panel. Scale bar; 100 µm. **H** Immunostaining of active-OPN in saline-treated, BoTA-treated, and BoTA with IGFBP3 neutralizing antibody-treated thin endometrium. Upper panel shows the expression of active-OPN in the whole region of uterus and the indicated regions (*LE* luminal epithelium, *GE* glandular epithelium) with boxes are magnified in lower panel. Scale bar: 100 µm. **I** H&E images for saline-, BoTA-, and BoTA with IGFBP3 neutralizing antibody-treated uteri. Scale bar: 100 µm

We next examined the molecular mechanisms underlying the recovery of thin endometrium via intrauterine administration of BoTA in an experimentally-induced thin endometrium mouse model. Our interrogation of DEG analyses discovered that the *Igfbp3* gene is shared between the two groups, including factors classified in the CC GO terms derived from EtOH- (GSE207379) or BoTA-treated (GSE146934) uteri compared to saline-treated controls (Figs. 3C, 5A, and Supplemental Fig. S3B, and, Table S2). Moreover, genes involved in the CC category concordantly interacted with *Spp1* (OPN), which we found as a key regulator in the thin endometrium of both mice and humans (Figs. 3C, 5B, and Supplemental Fig. S3B). To support this notion, immunoblotting analyses displayed that intrauterine BoTA administration re-elevated the decreased levels of both IGFBP3 and the 35 kDa-OPN observed in the EtOH-induced thin endometrium (Fig. 5C and Supplemental Fig. S5A–C). This recovery effect on OPN proteolytic cleavage event generating increased expression of 35 kDa-OPN fragment was also observed with intrauterine administration of mouse recombinant IGFBP3 protein (Fig. 5D and Supplemental Fig. S5D). In order to test whether BoTA-induced proteolytic cleavage of OPN was mediated by elevated IGFBP3 via BoTA administration, an IGFBP3 neutralizing antibody (3 μg/ml) was applied together with BoTA (2 IU) into the uterine cavity of an EtOH-induced thin endometrium to counteract BoTA-mediated IGFBP3 induction (Fig. 5E). Immunoblotting analyses confirmed that IGFBP3 suppression was successfully mediated by the application of a neutralizing antibody. Interestingly, we found that a reduction in BoTA-induced proteolytic cleavage of OPN was accompanied with suppression of IGFBP3 (Fig. 5F and Supplemental Fig. S5E, F), which was consistently observed with the immunostaining of IGFBP3 and active-OPN (Fig. 5G–H and Supplemental Fig. S5G, H), implicating the molecular mechanism underlying BoTA-mediated induction of IGFBP3-dependent OPN proteolytic cleavage. In addition, suppression of IGFBP3 mediated by a neutralizing antibody diminished the therapeutic effects of intrauterine BoTA administration on the recovery of endometrial thickness in EtOH-induced thin endometrium (Fig. 5I and Supplemental Fig. S5I, J). These findings support that IGFBP3-mediated elevation of active-OPN is facilitated by BoTA treatment and this might be a key molecular mechanism of BoTA-mediated enhancement of uterine repair in the thin endometrium.

### No reproductive toxicity of intrauterine BoTA administration in mice

**Fig. 6:**
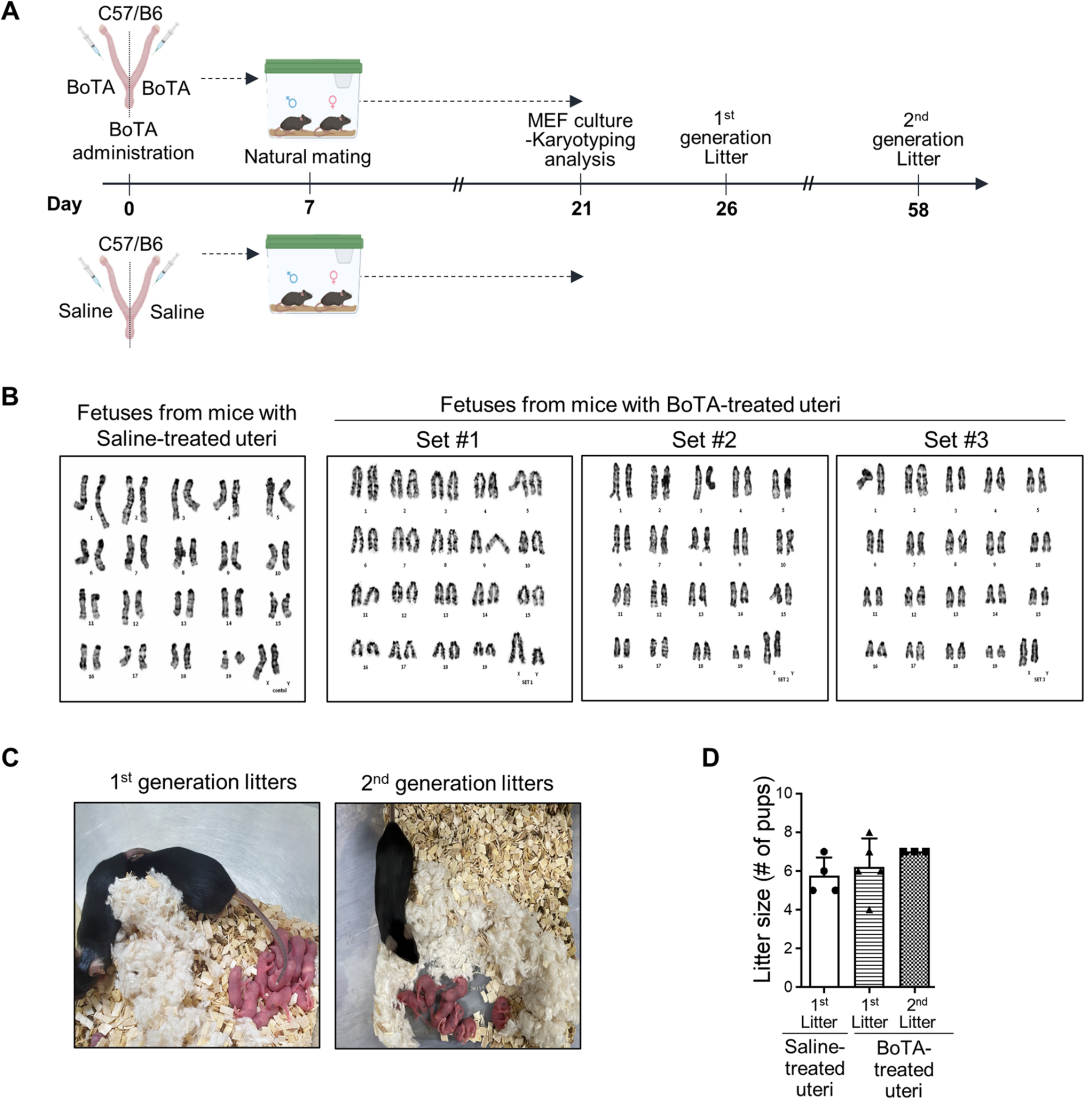
No reproductive toxicity of intrauterine BoTA administration in mice **A** An experimental time-line for MEFs generation and karyotyping analyses. **B** Karyotype analyses using MEFs derived from fetuses from mice with BoTA-treated uteri compared to those from saline-treated uteri. **C** Representative images of mouse mothers and their litters at 0–48 h after birth. Left panel: first generation, Right panel: second generation. **D** The number of offspring of the 1st and 2nd generation compared to normal mouse and data are expressed as mean ± SD, analyzed using the ordinary one-way ANOVA with Dunnett’s multiple comparisons test including *P* values (*< 0.05, **< 0.01, ***< 0.001, ****< 0.0001, *NS* not significant). *MEF*s mouse embryonic fibroblasts

We finally performed a reproductive toxicity test using a karyotyping analysis to determine if intrauterine BoTA administration induced any teratogenic effects in the subsequent generations. Mouse embryonic fibroblasts (MEFs) derived from day 13.5 post-coitum fetuses of mice with BoTA-treated uteri were used for karyotyping analyses (Fig. 6A). It revealed that no karyotypic abnormalities or chromosomal aberrations were detected in MEFs obtained from the fetuses derived from mice with BoTA-treated uteri compared to those from saline-treated uteri and mouse chromosomal references (Fig. 6B). Furthermore, no significant morphological (observed up to 5-week old) and numerical differences in both 1st and 2nd generation litters born from mice with saline- and BoTA-treated uteri were detected (Fig. 6C–D).

## Discussion

In the present study, a murine model of thin endometrium was successfully established by demonstrating dramatically decreased endometrial thickness and embryo implantation rates compared to those observed in saline-treated normal endometrium displaying a significant reduction in IGFBP3 and an active 35 kDa-form of OPN expression, which we consider as key molecular features of thin endometrium in both mice and humans. Upon intrauterine administration of BoTA, compromised endometrial thickness and fertility rates of our thin endometrium murine model were significantly restored displaying increased levels of endometrial receptivity and angiogenesis. Furthermore, BoTA administration recovered the expression levels of IGFBP3 and increased proteolytic cleavage of OPN re-generating the expression of the 35 kDa-OPN, and neutralization of BoTA-induced IGFBP3 suppressed proteolytic cleavage of OPN as observed in EtOH-induced thin endometrium, implicating that BoTA-induced recovery of the uterine environment might be mediated by IGFBP3-dependent OPN proteolytic cleavage.

A thin endometrial thickness less than 7 mm is a common etiological condition of the endometrium that affects the successful outcome of embryo implantation and pregnancy maintenance [9, 43]. There are currently no evidence-based effective treatments that are used in clinics to improve the structural and functional morphology of the thin endometrium [43]. Our current study demonstrates that an experimentally-induced mouse model of thin endometrium exhibits pathophysiological features including remarkably reduced thickness of the endometrium, decreased expression of endometrial receptivity- and angiogenesis-related genes, aberrantly reduced proliferative and angiogenic cellular capacity, and increased fibrotic lesions, which were lasted for the next 12 days, approximately three estrous cycles. Importantly, these conditions are consistently observed in human endometrial tissues obtained from patients with chronic thin endometrium [9]. Instillation of ethanol was aimed to induce long-lasting and broad damage in endometrial tissues by causing dehydration and denaturation of proteins resulting in similar pathophysiological features of chronic thin endometrium, synechia, intrauterine adhesions or fibrosis following damage to the basal layer of the endometrium [9, 37, 44]. This strongly suggests the successful establishment of a reproducible and reliable murine model with thin endometrium that can be usefully utilized for the research on improving the rates of embryo implantation and pregnancy in patients with a thin endometrium.

In further, we evaluated the therapeutic effect of BoTA intrauterine administration on thin endometrium using our murine thin endometrium model. This revealed that BoTA infusion significantly induced endometrial regeneration, displaying increased endometrial thickness and reduced fibrotic lesion, leading to elevated rates of embryo implantation via improved endometrial receptivity and angiogenesis in the damaged endometrium. In particular, vasculogenesis and angiogenesis are crucial during implantation and successful pregnancy. The angiogenic effects of BoTA have been recently reported by demonstrating that BoTA treatment improved the long-term retention of autologous fat grafting via augmented vascularization [45] and promoted blood flow during perfusion of cutaneous and myocutaneous flaps [46, 47]. In addition, BoTA injection enhanced neural regeneration in a murine model of tibial nerve neurotmesis [48], and intradermal injection of BoTA restored hair growth and regenerated hair follicle cells via suppression of neurogenic inflammation [49, 50]. Furthermore, we previously suggested the extension of BoTA use indication to the uterus with the aim of enhancing fertility, demonstrating its effectiveness in elevating endometrial angiogenesis and receptivity [26]. This study also suggests that sufficiently induced endometrial angiogenesis plays a critical role in re-constituting the endometrium as it becomes more receptive. Given these findings, we hypothesized that BoTA administration might have beneficial therapeutic effects on endometrial regeneration of the thin endometrium which is characterized mainly by insufficient endometrial growth, low expression of VEGF, and high blood flow impedance [9]. In this aspect, we conducted and evaluated the therapeutic effect of BoTA intrauterine administration on thin endometrium using our murine model revealing the improvement of endometrial environment via a significant increase in blood supply. These results suggest that BoTA could be a new adjuvant therapy to increase the rates of embryo implantation by improving the uterine environment. Moreover, studies have reported that BoTA has an anti-inflammatory effect as it decreases the expression of IL-1β or TNF-α in the ankle synovial tissues [47, 51], which is consistent with our findings. BoTA was positively involved in the recovery of the uterine environment by reducing fibrosis and inflammation-related factors after infusion into the damaged endometrium. The clinical use of BoTA has not been limited to the field of facial aesthetics and pain management based on muscle relaxation. However, over the past 20 years it has been expanded extensively to the various fields including cancer, depression, and gynecological disease [52–54]. Moreover, its safety in the human body has already been proven in numerous medical fields by reporting the remarkably effective and safe results of BoTA treatment for the cosmetic purposes, obtained from 1474 subjects and quantitatively safe and tolerable profiles of BoTA utilization from a meta-analysis involving 2309 subjects [55]. Consistent with these previous reports, our evaluation of the safety of intrauterine administration of BoTA revealed no cellular or reproductive toxicity observed in mice.

Functional enrichment analyses of GO terms in the comparison of EtOH-treated endometrium with saline-treated control group revealed that gene alterations in EtOH-induced thin endometrium were mainly related to lipid metabolic processes, such as “triglyceride metabolic process”, “regulation of glycogen biosynthetic process”, and “arachidonate 12-lipoxygenase activity”. Fatty acids and their metabolites are well known to be very important in all stages of pregnancy and support cell proliferation and vascularization [56]. Triglycerides and eicosanoids, lipid mediators secreted from the endometrium affect the endometrial receptivity via producing prostaglandins, thromboxanes, and steroid hormones including estrogen and progesterone [57]. Additionally, transcriptome analyses of human thin endometrial tissues identified the down-regulated genes those are involved in catabolic processes, which are essential for breaking down of large molecules, such as polysaccharides and lipids into smaller units [40]. These results suggest that cellular energy synthesis might be significantly down-regulated in the EtOH-treated thin endometrium, similar to that in the human thin endometrium. Furthermore, GO term analyses revealed that enriched genes of EtOH-treated endometrium were largely involved in immunity, including “cellular response to interleukin-1 and 13”, “immune system process”, and “cytokine production”. Aberrant immunological factors are reported to be responsible for repeated implantation failure and 56.6% of the patients who experienced embryo implantation failures showed local immune over-activation in the endometrium at the mid‐luteal phase [58]. This might be the supporting evidence that EtOH-induced thin endometrium recapitulates aberrant immunological features of the human thin endometrium.

Furthermore, cellular dynamics to support the uterine regeneration are essentially required for the endometrium to recover during the menstrual cycle, which is weakly manifested in the thin endometrium resulting in insufficient endometrial growth [9]. This is consistently observed in our RNA-seq analyses displaying that top-ranked ontology terms are significantly involved in “extracellular region”, “apical plasma membrane”, “insulin-like growth factor ternary complex”. In particular, *Igfbp3* was identified as a shared enriched gene in DEG analyses of the EtOH-induced thin endometrium and BoTA-treated endometrium. This revealed *Igfbp3* was significantly down-regulated in the murine thin endometrium, whereas it was dramatically up-regulated by BoTA administration, which suggests that IGFBP3 can be considerable as a key regulator of BoTA-induced endometrial regeneration. IGFBP3 has been reported to play roles in tissue regeneration regulating cell proliferation, migration, and differentiation [59, 60]. Although little is known about the impact of BoTA on the regulation of IGFBP3, BoTA-induced angiogenic effects might be caused by increased IGFBP3 since knockdown of IGFBP3 has been reported to reduce sprouting angiogenesis in a 3-dimensional microfluidic angiogenesis model using human umbilical endothelial cells [61]. Moreover, transcriptome analyses of the receptive endometrium during embryo implantation in goats revealed that IGFBP3 is strongly involved in the endometrial proliferation and growth [62], which is consistent with our findings demonstrating increased IGFBP3 was detected in functionally and structurally recovered endometrium following BoTA intrauterine administration.

The gene–gene interaction network identified a shared gene “*Spp1* (OPN)” among DEGs from GO terms of cellular components enriched in EtOH-treated endometrium compared to the control. It has been shown that OPN has critical roles in the adhesion and migration of various cell types by interacting with different cell surface receptors of the integrin family, primarily integrin αvβ3 [63]. In the endometrium, OPN is expressed in both the glandular and luminal epithelium during the mid to late secretory phase of the menstrual cycle under the regulation of progesterone and estrogen [64]. The integrin αvβ3-OPN interaction is reported to be important for the process of embryo implantation [29]. Crucially, our immunoblotting analyses revealed that the cleaved-OPN fragment was clearly observed in normal endometrial tissues whereas OPN is failed to undergo the cleavage event in EtOH-induced murine thin endometrium. OPN contains several cleavage sites close to its integrin-binding motifs [65, 66]. Thus, proteolytic cleavage of OPN might mediate greater exposure to various ligands, especially integrin αvβ3, and lead to higher rates of molecular interaction. It is abundantly present at the apical surfaces of the endometrial glandular epithelium during the early stage of embryo implantation and initiates the process of embryo attachment by interacting with integrin proteins [67]. Among the many cleaved forms, the 35 kDa-fragment (active-OPN) has been mainly described as being cleaved by thrombin and is known to be more stimulatory to cell attachment and migration than the 70 kDa-fragment, native-OPN [67]. In our results, a significant decrease in the 35 kDa-form of OPN was observed in the thin endometrium of our mouse model, which was dramatically restored back to the levels in the normal endometrium by BoTA treatment displaying clear localization in the luminal and glandular epithelium. Based on these results, we inferred that BoTA administration might induce thrombin-mediated proteolytic cleavage of OPN within the direct or indirect association of various factors involved in the plasma membrane, extracellular region, and insulin-like growth factor complex leading to stronger and more stable cell–cell and cell–matrix adhesion, which is eventually resulting in enhancement of the rates of embryo implantation.

Significantly, we demonstrated that BoTA administration recovered the damaged structure and impaired function of the thin endometrium by increasing cellular proliferation and vessel formation, and reducing collagen accumulated lesions, resulting in improved embryo implantation. We propose that the failure of proteolytic cleavage of OPN might be a key molecular feature of thin endometrium, which we found was fully recovered by increased IGFBP3 via BoTA administration. Further studies addressing the molecular relevance of the BoTA treatment and IGFBP3-dependent proteolytic cleavage of OPN in the regulation of endometrial regeneration and embryo implantation may further aid in supporting the efficacy and safety of BoTA for broader applications in patients with thin endometrium.

### Supplementary Information

Below is the link to the electronic supplementary material.

Supplementary file1 (DOCX 12878 KB)

## Data Availability

RNA-seq data that support the findings of this study have been deposited in GEO with the primary accession code GSE207379. The authors declare that all other data supporting the findings of this study are available within the article and its Supplementary information files.
