# Supplementary material for: Intrauterine botulinum toxin A administration promotes endometrial regeneration mediated by IGFBP3-dependent OPN proteolytic cleavage in thin endometrium
Source: Cell Mol Life Sci. 2023 Jan 5;80(1):26. doi: 10.1007/s00018-022-04684-6 (PMC9816300; doi:10.1007/s00018-022-04684-6)
Supplement: Supplementary file 1 — Supplementary file1 (DOCX 12878 KB) [file 18_2022_4684_MOESM1_ESM.docx]

**Intrauterine Botulinum Toxin A administration promotes endometrial regeneration mediated by IGFBP3-dependent OPN proteolytic cleavage in thin endometrium**

^1^Danbi Lee, ^2^Jungho Ahn, ^3, #^Hwa Seon Koo, ^2, #^Youn-Jung Kang

**Affiliations**

^1^Department of Biomedical Science, School of Life Science, CHA University, 335 Pangyo-ro, Bundang-gu, Seongnam-si, Gyeonggi-do, South Korea

^2^Department of Biochemistry, Research Institute for Basic Medical Science, School of Medicine, CHA University, 335 Pangyo-ro, Bundang-gu, Seongnam-si, Gyeonggi-do, South Korea

^3^CHA Fertility Center Bundang, 59, Yatap-ro, Bundang-gu, Seongnam-si, Gyeonggi-do, South Korea

**^#^Corresponding Authors:** Youn-Jung Kang ([yjkang@cha.ac.kr](mailto:yjkang@cha.ac.kr)), Hwa Seon Koo (hwas0605@cha.ac.kr),


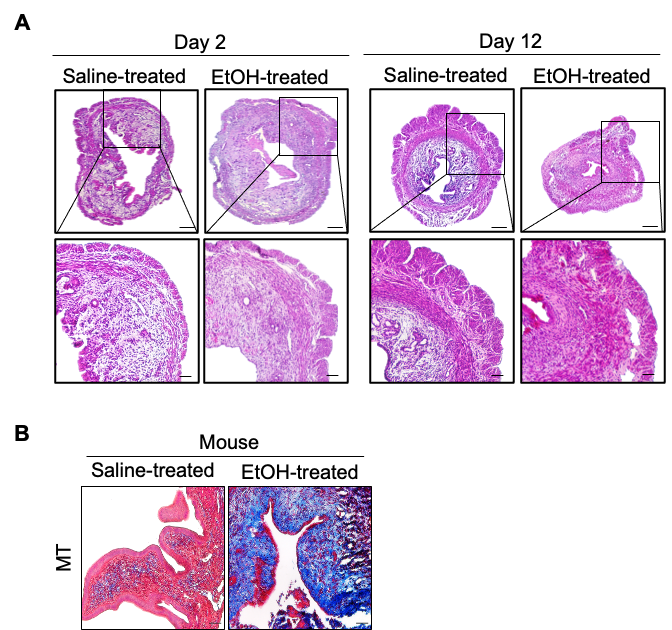


**Supplemental Figure S1.**

**(A)** Representative 2 H&E images of 95% EtOH-treated vs. saline-treated uteri harvested on Day 2 and 12. Upper panel shows the entire region of each uterus and magnified images are shown in lower panel. **(B)** Comparison of deposition of Collagen in endometrial tissues in mouse samples (Saline-treated vs. EtOH-treated) by MT staining

**
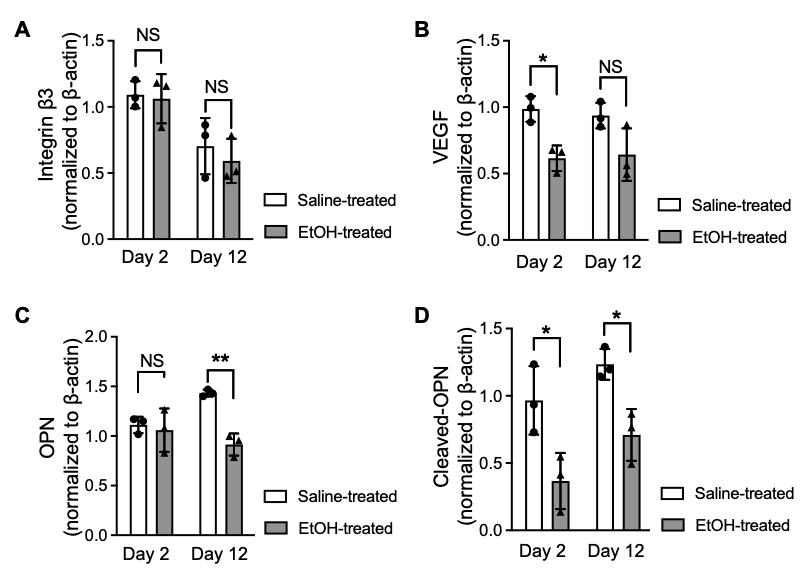
**

**Supplemental Figure S2.**

Densitometry of immunoblot bands of integrin β3 **(A)**, VEGF **(B)**, OPN **(C)**, and cleaved-OPN **(D)** shown in Figure 2 using Image J. Loading control; β-actin. 3 independent experiments were performed. Data shown in **(A-D)** are analyzed by two-way ANOVA with Tukey’s multiple comparisons test including P-values (*<0.05, **<0.01, ***<0.001, ****<0.0001).

**
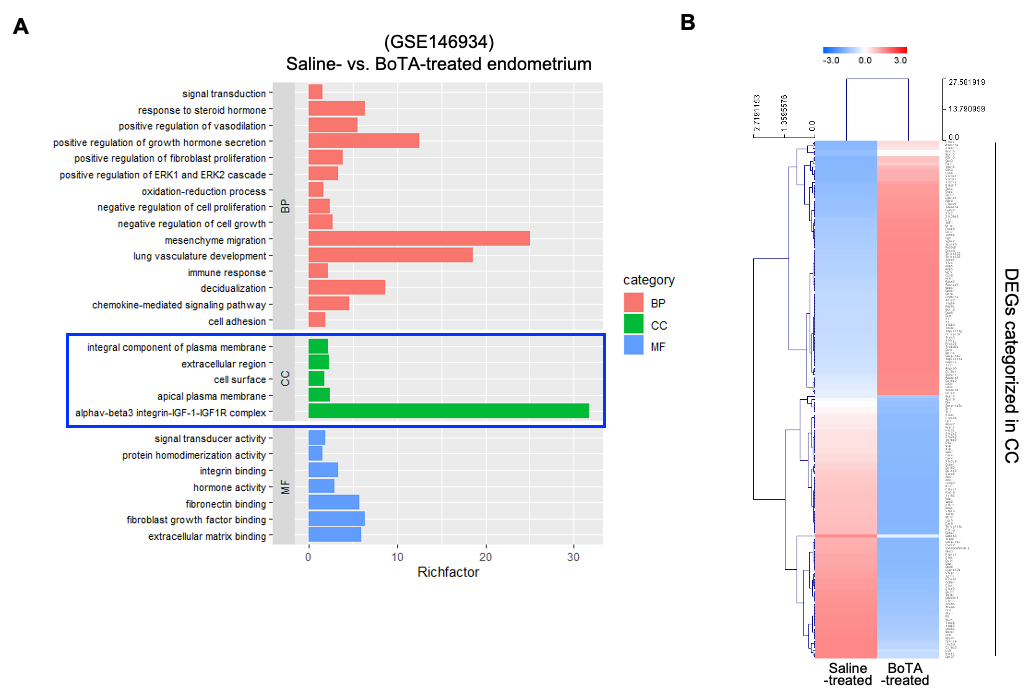
**

**Supplemental Figure S3.**

**(A)** Bar chart displaying biological process (BP), cellular component (CC), molecular function (MF) of gene ontology (GO) and pathway analysis of differentially expressed from GSE146934 using DAVID tool and their fold enrichment on X-axis. Chart was plotted by R studio. **(B)** Heatmap of DEGs from cellular component (CC) terms shown in **(A)**.

**
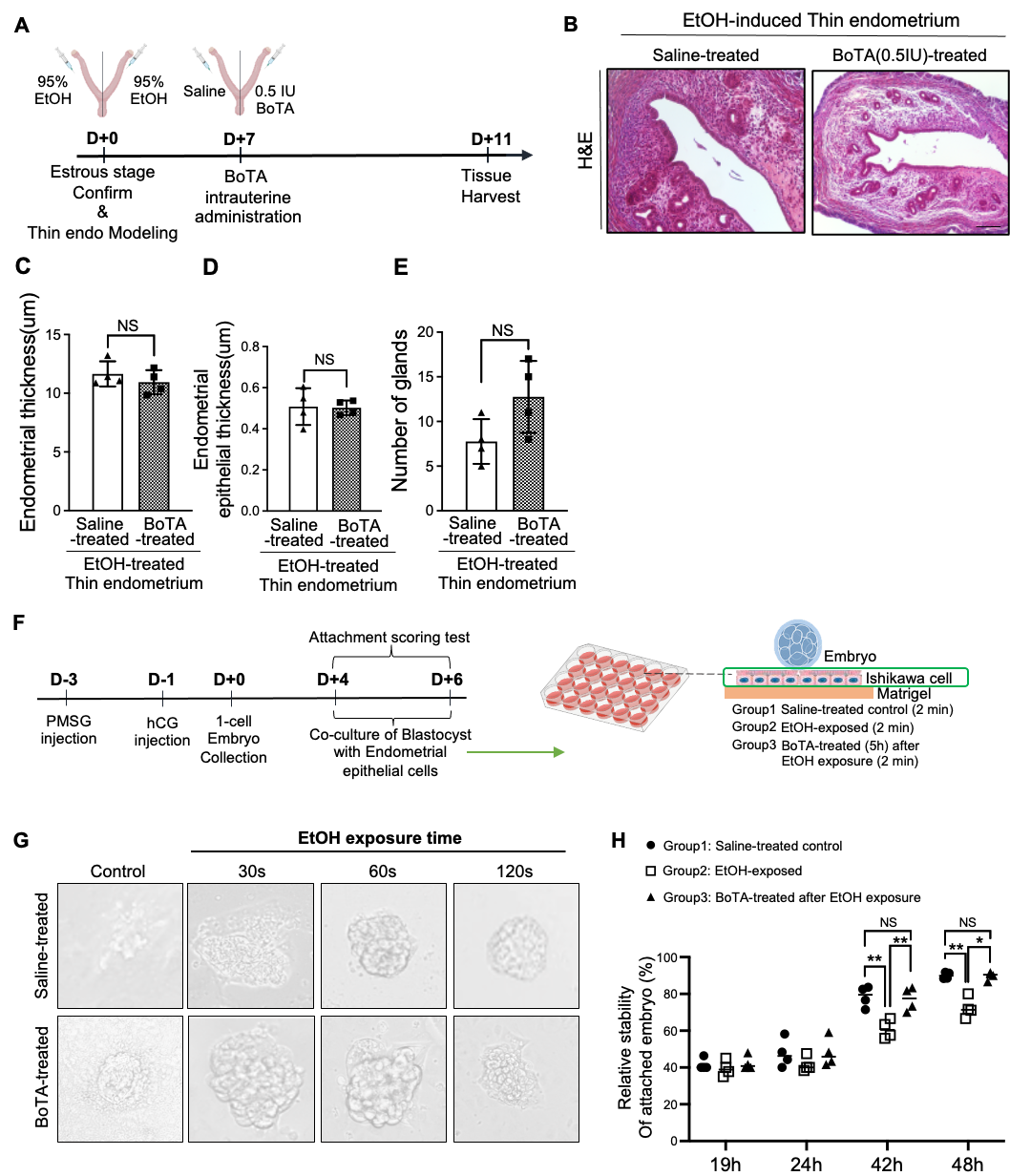
**

**Supplemental Figure S4.**

**(A)** An experimental schedule for intrauterine administration of 0.5 IU BoTA. **(B)** H&E staining of 0.5 IU BoTA-treated thin endometrium compared to saline-treated thin endometrium. Scale bar; 100 µm. **(C-E)** Comparisons of endometrial epithelial thickness, endometrial thickness and total number of glands between saline-treated thin endometrium and 0.5 IU BoTA-treated thin endometrium. Data are expressed as mean ± SD and analyzed by unpaired t test including P-values (*<0.05, **<0.01, ***<0.001, ****<0.0001, NS; not significant). **(F)** An experimental schedule and design for the embryo attachment scoring test. **(G)** Representative images of attached embryos onto EtOH-primed (indicated exposure time) saline-treated or BoTA-treated Ishikawa cells at 48h after co-culture. **(H)** Relative stability of attached embryos in each condition. Data shown for **(H)** are from 3 independent experiments (9 of mice, 95 of embryos) and analyzed by two-way ANOVA with Tukey’s multiple comparisons test including P-values (*<0.05, **<0.01, ***<0.001, ****<0.0001).

**
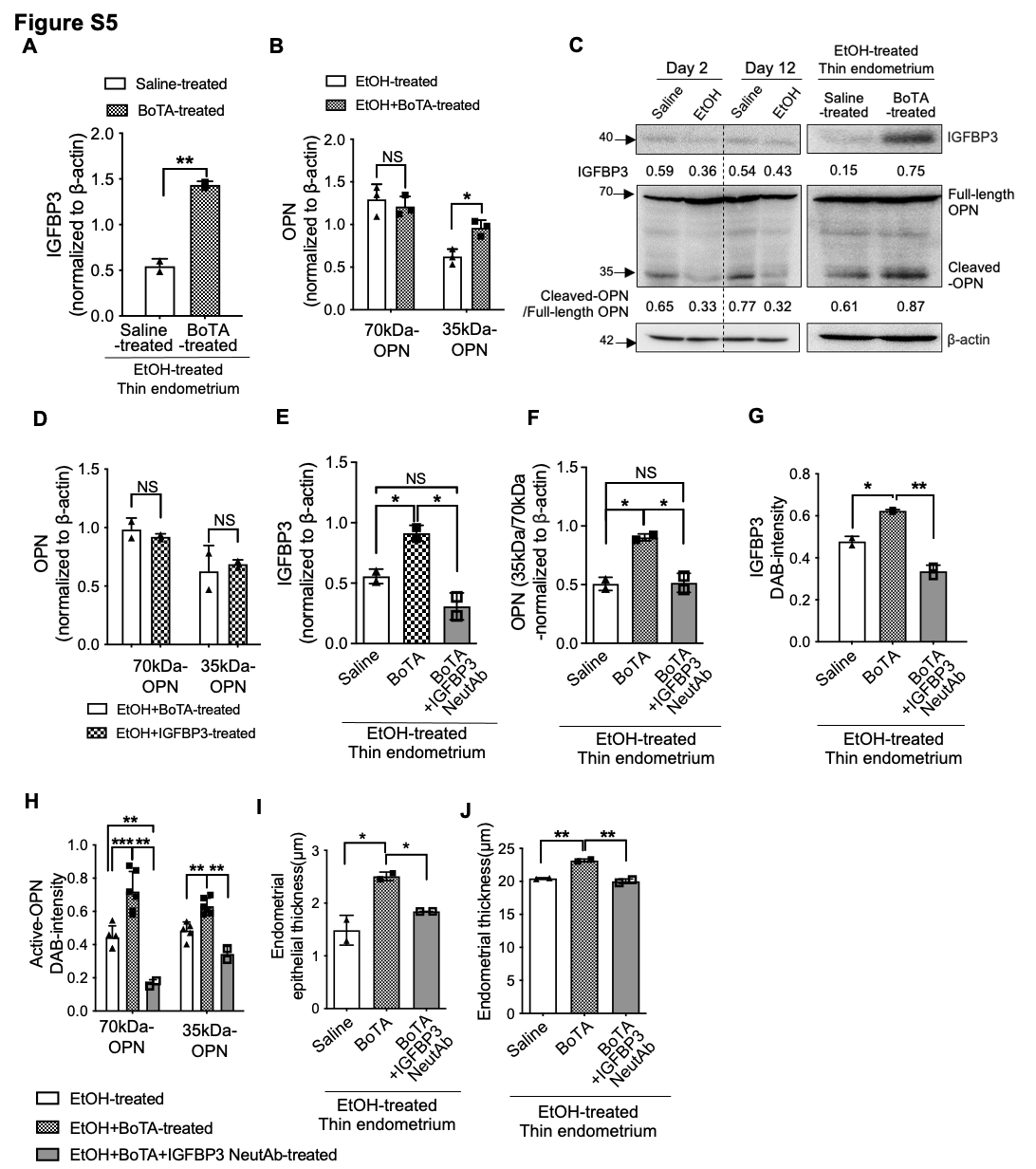
**

**Supplemental Figure S5.**

Densitometry of immunoblot bands of IGFBP3 **(A)**, OPN **(B)** shown in **Figure 5C** using Image J. Loading control; β-actin. 3 independent experiments were performed. Data shown in **(A)** are expressed as mean ± SD and analyzed using the two-way ANOVA with Tukey’s multiple comparisons test, and including P-values (*<0.05, **<0.01, ***<0.001, ****<0.0001, NS; not significant). Data shown in **(B)** are analyzed using the Unpaired t test. **(C)** Immunoblotting analysis of IGFBP3 and OPN in saline-treated vs. EtOH-treated (Day 2 & 12) and saline-treated vs. BoTA-treated thin endometrium. Numbers below each blot for IGFBP3 are corresponding to the densitometry of each band, which was normalized to the β-actin, and numbers below each blot for OPN are corresponding to the densitometry of each band of cleaved-OPN, which was normalized to the β-actin and then standardized to Full-length OPN. **(D)** Densitometry of immunoblot band of OPN shown in Figure 5D using Image J. Loading control; β-actin. **(E-F)** Densitometry of immunoblot band of IGFBP3 and OPN (35kDa/70kDa) shown in Figure 5F using Image J. Loading control; β-actin. IHC images shown in **Figure 5G-H** are quantified in graphs in **(G-H)**. Data shown in **(G)** are analyzed using the ordinary one-way ANOVA with Dunnett’s multiple comparisons test, and data shown in **(H)** are expressed as mean ± SD and analyzed using the two-way ANOVA with Tukey’s multiple comparisons test, and including P-values (*<0.05, **<0.01, ***<0.001, ****<0.0001, NS; not significant). Quantification of histological evaluations of endometrial epithelial thickness **(I)**, endometrial thickness **(J)** shown in **Figure 5I** and data shown in **(I-J)** are analyzed using the ordinary one-way ANOVA with Dunnett’s multiple comparisons test including P-values (*<0.05, **<0.01, ***<0.001, ****<0.0001, NS; not significant).

**Supplemental Figure S6.**

Whole blot of Figure 2E

**Supplemental Figure S7.**

Whole blot of Figure 5C **(A)**, 5D **(B)**, 5F **(C)**

| **Species** | **Gene** | **Direction** | **Sequence** |
| --- | --- | --- | --- |
| **Mouse** | *Rpl7* | Forward | TCAATGGAGTAAGCCCAAAG |
|  |  | Reverse | CAAGAGACCGAGCAATCAAG |
|  | *Spp1* | Forward | AGAGCGGTGAGTCTAAGGAG |
|  |  | Reverse | TGCCCTTTCGGTTGTTGTCC |
|  | *Vegfb* | Forward | TGACATCATCCATCCCACTC |
|  |  | Reverse | CCTTGGCAATGGAGGAAG |
|  | *Vegfa* | Forward | GCACATAGAGAGAGAATCAGCTTC |
|  |  | Reverse | CTCCGCTCTGAACAAGGCT |
|  | *Tie1* | Forward | GGTCACACACACGGTGAACAA |
|  |  | Reverse | TGCCAGTCTAGGGTATTGAAG |
|  | *Ang1* | Forward | AAAATGGGTTTTGGGAATCCC |
|  |  | Reverse | TCGGCACCGTGTAAGATCAAG |
|  | *Vegfr2* | Forward | CCACACGAGGCGTGAACTC |
|  |  | Reverse | CTTCAGGTTACATCGCGGTGA |
|  | *Il-1b* | Forward | GAAATGCCACCTTTTGACAGTG |
|  |  | Reverse | TGGATGCTCTCATCAGGACAG |
|  | *Tgfb1* | Forward | GTGAAACGGAAGCGCATCGAAG |
|  |  | Reverse | CATAGTAGTCCGCTTCGGGCTCC |
|  | *Timp1* | Forward | GACCTATAGTGCTGGCTGTGG |
|  |  | Reverse | GTAGTCCTCAGAGCCCACGA |
| **Human** | *ACTB* | Forward | CATGTACGTTGCTATCCAGGC |
|  |  | Reverse | GCCTTAATGTCACGCACGAT |
|  | *TGFB1* | Forward | AGAGTGTCTGCGGATACTTCC |
|  |  | Reverse | CCAACAGTGTAGGTCTTGGTG |
|  | *TIMP1* | Forward | CTAATGGTGGAAACCCACAACG |
|  |  | Reverse | TATCGCCAGGAATTGTTGCTG |
|  | *SPP1* | Forward | GAAGTTTCGCAGACCTGACAT |
|  |  | Reverse | GTATGCACCATTCAACTCCTCG |

**Supplemental Table S1.** Primer sequence pairs used for RT-PCR analyses

| **Gene symbol**  **(Up-regulated)** | **P-values** | **Fold changes** |
| --- | --- | --- |
| Tceal8 | 0.019 | 2.032 |
| G530011O06Rik | 0.043 | 2.754 |
| Pi15 | 0.016 | 2.826 |
| Igf1 | 0.016 | 4.103 |
| 2210015D19Rik | 0.008 | 2.076 |
| Tmem17 | 0.010 | 2.106 |
| Nipal4 | 0.000 | 2.205 |
| Acsl6 | 0.005 | 3.760 |
| Pmp22 | 0.026 | 2.002 |
| Rangrf | 0.031 | 2.220 |
| Kcnab3 | 0.004 | 2.742 |
| Ccl7 | 0.035 | 2.607 |
| Tmem132e | 0.008 | 2.170 |
| Tubd1 | 0.023 | 2.856 |
| Rad51b | 0.039 | 2.507 |
| Akr1c12 | 0.013 | 2.627 |
| Fam65b | 0.022 | 3.235 |
| Ear3 | 0.004 | 2.358 |
| Ear12 | 0.004 | 2.358 |
| Dok2 | 0.005 | 2.279 |
| Il7r | 0.005 | 2.141 |
| Tbc1d31 | 0.049 | 2.598 |
| Sqle | 0.046 | 2.733 |
| Khdrbs3 | 0.035 | 2.057 |
| C1qtnf6 | 0.016 | 2.220 |
| Lgals1 | 0.031 | 2.419 |
| Ccdc134 | 0.026 | 2.366 |
| Mettl22 | 0.004 | 2.265 |
| Vgll3 | 0.019 | 2.580 |
| Adamts1 | 0.006 | 2.574 |
| Runx1 | 0.024 | 2.395 |
| Zfp948 | 0.023 | 2.095 |
| Rab26os | 0.040 | 2.217 |
| Zfp119b | 0.007 | 2.516 |
| Tubb6 | 0.039 | 2.522 |
| 1810055G02Rik | 0.023 | 2.828 |
| Fxn | 0.004 | 2.172 |
| Slc16a12 | 0.018 | 2.523 |
| Ffar4 | 0.020 | 2.674 |
| Hacd1 | 0.016 | 3.352 |
| Lhx6 | 0.013 | 2.664 |
| Lrp4 | 0.030 | 2.264 |
| Rcn1 | 0.019 | 2.019 |
| Bfsp1 | 0.032 | 2.062 |
| Slc2a10 | 0.003 | 3.310 |
| Abhd16b | 0.014 | 2.646 |
| Dclk1 | 0.014 | 2.540 |
| Slitrk3 | 0.016 | 2.411 |
| Ctps | 0.031 | 2.516 |
| Ajap1 | 0.011 | 2.928 |
| Ccdc146 | 0.033 | 2.428 |
| Smarcd3 | 0.012 | 2.995 |
| Ndufa4 | 0.001 | 2.027 |
| Clec5a | 0.033 | 5.834 |
| Zfp248 | 0.024 | 2.911 |
| Mfap5 | 0.014 | 2.227 |
| Plaur | 0.008 | 2.638 |
| Fbxo17 | 0.011 | 2.239 |
| Ano5 | 0.038 | 2.565 |
| Mrps11 | 0.002 | 2.269 |
| Chrdl2 | 0.048 | 2.569 |
| Hbb-bt | 0.034 | 3.700 |
| Hbb-b2 | 0.034 | 3.728 |
| Hbb-bs | 0.005 | 3.938 |
| Hbb-b1 | 0.005 | 3.939 |
| Ifitm6 | 0.003 | 2.654 |
| Tnni2 | 0.011 | 2.843 |
| Ascl2 | 0.011 | 2.141 |
| Scarna9 | 0.026 | 2.283 |
| Thyn1 | 0.029 | 2.054 |
| Slc37a4 | 0.012 | 2.049 |
| Loxl1 | 0.016 | 2.412 |
| Bcl2a1a | 0.044 | 2.120 |
| Plod2 | 0.018 | 2.870 |
| Rnf113a1 | 0.004 | 2.360 |
| **Gene symbol**  **(Down-regulated)** | **P-values** | **Fold changes** |
| Paqr8 | 0.013 | 0.499 |
| Il1rl2 | 0.007 | 0.454 |
| Il18r1 | 0.045 | 0.442 |
| Mogat1 | 0.001 | 0.269 |
| Sp100 | 0.040 | 0.385 |
| 4933407L21Rik | 0.000 | 0.473 |
| Rab17 | 0.038 | 0.272 |
| Ikbke | 0.002 | 0.339 |
| Ptgs2os2 | 0.037 | 0.447 |
| Nos1ap | 0.041 | 0.499 |
| Ipcef1 | 0.002 | 0.473 |
| Pdxk | 0.011 | 0.236 |
| Misp | 0.036 | 0.322 |
| Cpm | 0.008 | 0.235 |
| Gck | 0.000 | 0.309 |
| Igfbp3 | 0.032 | 0.158 |
| 4930415F15Rik | 0.049 | 0.466 |
| Cobl | 0.044 | 0.386 |
| Tgtp2 | 0.050 | 0.458 |
| Slc22a4 | 0.034 | 0.359 |
| Alox12e | 0.006 | 0.238 |
| Alox15 | 0.008 | 0.249 |
| Shpk | 0.032 | 0.471 |
| Tmem98 | 0.023 | 0.354 |
| Hoxb2 | 0.028 | 0.490 |
| Plxdc1 | 0.044 | 0.429 |
| Fbxl20 | 0.013 | 0.383 |
| Abca8b | 0.038 | 0.463 |
| Abca8a | 0.039 | 0.281 |
| Cog1 | 0.018 | 0.433 |
| Gprc5c | 0.019 | 0.434 |
| BC100451 | 0.048 | 0.476 |
| Hgs | 0.039 | 0.435 |
| Hs1bp3 | 0.013 | 0.363 |
| Lrrn3 | 0.023 | 0.302 |
| Gm17821 | 0.025 | 0.492 |
| Dhrs7 | 0.018 | 0.475 |
| Syne2 | 0.035 | 0.489 |
| Vash1 | 0.000 | 0.438 |
| Clmn | 0.012 | 0.407 |
| D430020J02Rik | 0.016 | 0.460 |
| Tmem196 | 0.025 | 0.450 |
| Tubb2b | 0.001 | 0.272 |
| Mylip | 0.049 | 0.426 |
| S1pr3 | 0.025 | 0.303 |
| Chdh | 0.030 | 0.493 |
| Txndc16 | 0.017 | 0.397 |
| Haus4 | 0.026 | 0.447 |
| Gjb2 | 0.041 | 0.368 |
| Klf5 | 0.011 | 0.474 |
| Tspyl5 | 0.020 | 0.435 |
| Peg13 | 0.013 | 0.458 |
| Ly6e | 0.014 | 0.348 |
| Kifc2 | 0.009 | 0.450 |
| 1810021B22Rik | 0.029 | 0.456 |
| Tmem117 | 0.032 | 0.331 |
| Pou6f1 | 0.016 | 0.305 |
| Serpind1 | 0.045 | 0.366 |
| E130310I04Rik | 0.035 | 0.358 |
| Dnajc28 | 0.044 | 0.380 |
| Fndc1 | 0.026 | 0.415 |
| Unc93a | 0.020 | 0.315 |
| Syngap1 | 0.049 | 0.431 |
| Tapbp | 0.033 | 0.466 |
| H2-K2 | 0.042 | 0.490 |
| AA388235 | 0.003 | 0.269 |
| Ppt2 | 0.011 | 0.410 |
| Tnxb | 0.012 | 0.450 |
| H2-Q4 | 0.001 | 0.400 |
| A930015D03Rik | 0.000 | 0.232 |
| Rcan2 | 0.050 | 0.406 |
| Rasgrp3 | 0.041 | 0.473 |
| Npc1 | 0.036 | 0.499 |
| Lims2 | 0.019 | 0.342 |
| Pcdhgc4 | 0.030 | 0.455 |
| 1700120E14Rik | 0.009 | 0.344 |
| Sptbn2 | 0.004 | 0.425 |
| Tmem151a | 0.037 | 0.240 |
| Ifit2 | 0.028 | 0.308 |
| Pdlim1 | 0.020 | 0.464 |
| Frat2 | 0.010 | 0.443 |
| Pax2 | 0.022 | 0.490 |
| Pcgf6 | 0.026 | 0.470 |
| Tprn | 0.042 | 0.462 |
| Naif1 | 0.050 | 0.492 |
| Gyltl1b | 0.046 | 0.463 |
| Shc4 | 0.010 | 0.490 |
| Zfp661 | 0.021 | 0.205 |
| Gm14057 | 0.016 | 0.451 |
| Slc52a3 | 0.041 | 0.387 |
| Lbp | 0.029 | 0.248 |
| Zfp831 | 0.025 | 0.458 |
| Pcmtd2 | 0.014 | 0.494 |
| Lrrc31 | 0.020 | 0.416 |
| Fmo5 | 0.021 | 0.376 |
| Ptpn22 | 0.019 | 0.449 |
| Gstm1 | 0.038 | 0.420 |
| Tox | 0.048 | 0.434 |
| Ddx58 | 0.048 | 0.457 |
| Enho | 0.033 | 0.474 |
| Dnajc6 | 0.032 | 0.408 |
| Acot11 | 0.001 | 0.426 |
| Spata6 | 0.011 | 0.459 |
| Sesn2 | 0.043 | 0.423 |
| B930041F14Rik | 0.046 | 0.494 |
| Perm1 | 0.050 | 0.353 |
| Sema3e | 0.022 | 0.267 |
| Napepld | 0.007 | 0.421 |
| Wdr86 | 0.048 | 0.480 |
| Txk | 0.003 | 0.333 |
| Spink2 | 0.039 | 0.434 |
| Cldn4 | 0.005 | 0.498 |
| Dlx5 | 0.043 | 0.473 |
| Strip2 | 0.019 | 0.400 |
| Gimap3 | 0.014 | 0.467 |
| Fam188b | 0.006 | 0.381 |
| 2310040G24Rik | 0.023 | 0.467 |
| 1810044D09Rik | 0.037 | 0.432 |
| Klra15 | 0.017 | 0.273 |
| Klra18 | 0.003 | 0.371 |
| Klra4 | 0.014 | 0.352 |
| Klra12 | 0.010 | 0.338 |
| Klra21 | 0.008 | 0.396 |
| Klra8 | 0.030 | 0.413 |
| 8430419L09Rik | 0.008 | 0.392 |
| Itpr2 | 0.003 | 0.434 |
| Ttyh1 | 0.001 | 0.368 |
| Sipa1l3 | 0.030 | 0.416 |
| Fam71e1 | 0.004 | 0.357 |
| Ccdc114 | 0.033 | 0.390 |
| Tenm4 | 0.014 | 0.436 |
| Arntl | 0.012 | 0.369 |
| Tubgcp2 | 0.026 | 0.485 |
| Sirt3 | 0.014 | 0.400 |
| Evi5l | 0.004 | 0.435 |
| Lrrc8e | 0.038 | 0.399 |
| Irs2 | 0.015 | 0.332 |
| Gm16159 | 0.033 | 0.376 |
| Lonrf1 | 0.001 | 0.392 |
| Mylk3 | 0.021 | 0.313 |
| Gpt2 | 0.016 | 0.299 |
| Cx3cl1 | 0.003 | 0.442 |
| Lrrc36 | 0.010 | 0.436 |
| Casp4 | 0.022 | 0.498 |
| Barx2 | 0.016 | 0.233 |
| Tmem136 | 0.033 | 0.498 |
| Gm5617 | 0.035 | 0.434 |
| Slc24a1 | 0.041 | 0.473 |
| Acpp | 0.017 | 0.328 |
| Hyal1 | 0.001 | 0.353 |
| Cmtm8 | 0.020 | 0.435 |
| Ccdc120 | 0.030 | 0.458 |
| AW822252 | 0.011 | 0.483 |
| Arhgef6 | 0.002 | 0.419 |

**Supplementary Table S2.** A list of differentially expressed genes of EtOH-treated endometrial samples compared to saline-treated control, shown in a heatmap of Fig. 3B. The cutoff for significance was set by P-value < 0.05 and Fold change > 2.0 (up-regulated genes) and < 0.5 (down-regulated genes).

| **Gene symbol**  **(Up-regulated)** | **P-values** | **Fold changes** |
| --- | --- | --- |
| Adamts1 | 0.008 | 2.359 |
| Adamts4 | 0.030 | 2.465 |
| Adcyap1 | 0.009 | 11.496 |
| Adgrb3 | 0.008 | 5.277 |
| Agr2 | 0.033 | 2.769 |
| Aqp5 | 0.012 | 3.315 |
| Asic2 | 0.001 | 2.545 |
| Bmp8a | 0.047 | 20.515 |
| Bpifc | 0.042 | 4.539 |
| Btbd17 | 0.011 | 2.660 |
| Cav3 | 0.005 | 5.186 |
| Ccl11 | 0.032 | 13.107 |
| Ccl7 | 0.029 | 7.979 |
| Cd34 | 0.038 | 2.047 |
| Col6a1 | 0.007 | 2.371 |
| Col6a2 | 0.035 | 2.312 |
| Cpa6 | 0.043 | 2.201 |
| Crispld2 | 0.006 | 2.957 |
| Dscam | 0.015 | 5.365 |
| F2rl3 | 0.006 | 4.255 |
| Ffar4 | 0.018 | 3.136 |
| Fgl1 | 0.003 | 2.375 |
| Gdf6 | 0.029 | 3.718 |
| Grm1 | 0.007 | 3.117 |
| Gypa | 0.019 | 7.356 |
| Hrh1 | 0.049 | 2.954 |
| Hspa1a | 0.002 | 2.548 |
| Igf1 | 0.037 | 2.554 |
| Igsf5 | 0.033 | 2.165 |
| Il11 | 0.045 | 3.237 |
| Il27ra | 0.049 | 2.047 |
| Il4ra | 0.019 | 2.169 |
| Inhbb | 0.009 | 4.080 |
| Itgb3 | 0.002 | 2.894 |
| Jchain | 0.017 | 3.054 |
| Kcne4 | 0.041 | 2.083 |
| Kl | 0.001 | 5.498 |
| Lgals1 | 0.033 | 2.207 |
| Lif | 0.008 | 4.021 |
| Ly96 | 0.002 | 2.510 |
| Muc13 | 0.040 | 3.536 |
| Myrip | 0.004 | 3.175 |
| Nppc | 0.036 | 13.837 |
| Nrtn | 0.046 | 2.692 |
| Ogn | 0.004 | 2.826 |
| Oxtr | 0.016 | 5.309 |
| Pcdh11x | 0.007 | 3.552 |
| Pcsk9 | 0.001 | 3.386 |
| Pm20d1 | 0.025 | 5.100 |
| Prss23 | 0.035 | 2.304 |
| Serpina9 | 0.004 | 7.816 |
| Sgca | 0.040 | 4.560 |
| Sgms2 | 0.001 | 2.492 |
| Slc39a8 | 0.043 | 2.387 |
| Slc3a1 | 0.020 | 2.774 |
| Spock3 | 0.014 | 16.438 |
| Spon1 | 0.036 | 2.722 |
| Stc2 | 0.033 | 2.522 |
| Tcam1 | 0.016 | 4.782 |
| Tfrc | 0.038 | 2.911 |
| Tmem45a | 0.036 | 2.317 |
| Tmprss11a | 0.010 | 12.702 |
| Tmprss11g | 0.009 | 9.881 |
| Trabd2b | 0.004 | 2.976 |
| Trpc3 | 0.011 | 2.363 |
| Wfdc15a | 0.041 | 2.177 |
| **Gene symbol**  **(Down-regulated)** | **P-values** | **Fold changes** |
| 2300002M23Rik | 0.004 | 0.224 |
| Ano1 | 0.018 | 0.486 |
| Apoc2 | 0.003 | 0.197 |
| Apol6 | 0.025 | 0.415 |
| Bcan | 0.016 | 0.291 |
| Car4 | 0.001 | 0.028 |
| Car6 | 0.033 | 0.195 |
| Ccbe1 | 0.018 | 0.448 |
| Ccl4 | 0.035 | 0.494 |
| Cd163 | 0.005 | 0.231 |
| Clca3b | 0.003 | 0.227 |
| Clcn5 | 0.044 | 0.255 |
| Col6a5 | 0.023 | 0.338 |
| Cthrc1 | 0.006 | 0.481 |
| Ctss | 0.040 | 0.429 |
| Cyp4a12a | 0.021 | 0.260 |
| Daw1 | 0.011 | 0.248 |
| Epha7 | 0.013 | 0.291 |
| Eppin | 0.015 | 0.247 |
| F9 | 0.041 | 0.350 |
| Flrt3 | 0.009 | 0.387 |
| Gabrb3 | 0.016 | 0.325 |
| Ghr | 0.018 | 0.498 |
| Ghrl | 0.043 | 0.375 |
| Gkn3 | 0.026 | 0.329 |
| Gldn | 0.048 | 0.222 |
| Gpc6 | 0.018 | 0.237 |
| Htr2a | 0.040 | 0.471 |
| Igf1r | 0.045 | 0.466 |
| Itih2 | 0.030 | 0.242 |
| Kcnh5 | 0.021 | 0.205 |
| Kiss1 | 0.025 | 0.312 |
| Klk11 | 0.042 | 0.384 |
| Klkb1 | 0.020 | 0.398 |
| Lgr5 | 0.001 | 0.146 |
| Lhb | 0.001 | 0.234 |
| Lpl | 0.015 | 0.246 |
| Mbl2 | 0.019 | 0.265 |
| Mip | 0.047 | 0.364 |
| Ms4a1 | 0.027 | 0.298 |
| Mup12 | 0.018 | 0.438 |
| Ndp | 0.034 | 0.114 |
| Nox4 | 0.012 | 0.236 |
| Npy2r | 0.023 | 0.264 |
| Ntrk1 | 0.031 | 0.391 |
| Olr1 | 0.012 | 0.427 |
| Oosp2 | 0.029 | 0.243 |
| Opn1sw | 0.041 | 0.313 |
| Plxnb1 | 0.048 | 0.479 |
| Ptprz1 | 0.045 | 0.099 |
| S1pr3 | 0.043 | 0.469 |
| Serpina3c | 0.033 | 0.279 |
| Serpine2 | 0.009 | 0.360 |
| Sfrp1 | 0.047 | 0.401 |
| Slc2a2 | 0.039 | 0.344 |
| Slc7a9 | 0.035 | 0.362 |
| Spink2 | 0.027 | 0.339 |
| Stc1 | 0.018 | 0.447 |
| Stra6 | 0.024 | 0.275 |
| Tgfbi | 0.015 | 0.326 |
| Them6 | 0.034 | 0.419 |
| Tnfrsf11b | 0.001 | 0.323 |
| Trf | 0.016 | 0.337 |
| Trpm1 | 0.008 | 0.267 |
| Vwa2 | 0.012 | 0.461 |
| Vwa7 | 0.020 | 0.437 |
| Wif1 | 0.003 | 0.006 |

**Supplementary Table S3.** A list of differentially expressed genes of BoTA-treated endometrial samples compared to saline-treated control, shown in a heatmap of Fig. S3B. The cutoff for significance was set by P-value < 0.05 and Fold change > 2.0 (up-regulated genes) and < 0.5 (down-regulated genes).

| **EtOH-treated vs. Saline-treated**  **(GSE207379)** | | **BoTA-treated vs. Saline-treated**  **(GSE146934)** | | **Shared** | | |
| --- | --- | --- | --- | --- | --- | --- |
| **Up-regulation** | **Down-regulation** | **Up-regulation** | **Down-regulation** | **Up-regulation** | **Down-regulation** | **Reciprocal** |
| C1qtnf6 | Acpp | Adamts1 | 2300002M23Rik | Adamts1 | S1pr3 | **Igfbp3** |
| Adamts1 | Ajap1 | Adcyap1 | Adamts4 | Ccl7 | Spink2 |  |
| Ccdc134 | Casp4 | Adgrb3 | Col6a5 | Ffar4 |  |  |
| Ccl7 | Cldn4 | Agr2 | Apol6 | Igf1 |  |  |
| Chrdl2 | Cx3cl1 | Aqp5 | Apoc2 | Lglas1 |  |  |
| Fam65b | Enho | Asic2 | Cpa6 |  |  |  |
| Ffar4 | Gstm1 | Bace2 | Ano1 |  |  |  |
| Igf1 | Hyal1 | Btbd17 | Bcan |  |  |  |
| Lgals1 | Igfbp3 | Bpifc | Cd8b1 |  |  |  |
| Loxl1 | Itpr2 | Clec2f | Bmp8a |  |  |  |
| Mfap5 | Lbp | Cav3 | Clca3b |  |  |  |
| Plaur | Nos1ap | Ccl11 | Clcn5 |  |  |  |
| Sema3e | Npc1 | Crispld2 | Car4 |  |  |  |
|  | Pi15 | Ccl7 | Car6 |  |  |  |
|  | S1pr3 | Cd34 | Ccbe1 |  |  |  |
|  | Sipa1l3 | Dpp6 | Col6a2 |  |  |  |
|  | Slc22a4 | Dscam | Cthrc1 |  |  |  |
|  | Slc52a3 | F2rl3 | Ctss |  |  |  |
|  | Spata6 | Fbln2 | Ccl4 |  |  |  |
|  | Spink2 | Ffar4 | Cxcr2 |  |  |  |
|  | Sptbn2 | Fgl1 | Col6a1 |  |  |  |
|  | Syne2 | Gdf6 | Cd163 |  |  |  |
|  | Tnxb | Gpx3 | Cd200r1 |  |  |  |
|  | Txndc16 | Grm1 | Cyp4a12a |  |  |  |
|  | Vash1 | Gypa | Epha7 |  |  |  |
|  |  | Igf1 | Eppin |  |  |  |
|  |  | Igfbp3 | F9 |  |  |  |
|  |  | Il11 | Flrt3 |  |  |  |
|  |  | ITGB3 | Fzd10 |  |  |  |
|  |  | Itgb6 | Gabrb3 |  |  |  |
|  |  | Jchain | Ghr |  |  |  |
|  |  | Kcne4 | Ghrl |  |  |  |
|  |  | Kl | Gkn3 |  |  |  |
|  |  | Lgals1 | Gldn |  |  |  |
|  |  | Lif | Gpc6 |  |  |  |
|  |  | Ly96 | Gpr37 |  |  |  |
|  |  | Muc13 | Hrh1 |  |  |  |
|  |  | Myrip | Hspa1a |  |  |  |
|  |  | Ngfr | Htr2a |  |  |  |
|  |  | Nppc | Igf1r |  |  |  |
|  |  | Nrtn | Il27ra |  |  |  |
|  |  | Ogn | Il4ra |  |  |  |
|  |  | Oxtr | Inhbb |  |  |  |
|  |  | Pcdh11x | Itga8 |  |  |  |
|  |  | Pcsk9 | Itih2 |  |  |  |
|  |  | Pm20d1 | Kcnh5 |  |  |  |
|  |  | Sgca | Kiss1 |  |  |  |
|  |  | Sgms2 | Klk11 |  |  |  |
|  |  | Slc3a1 | Klkb1 |  |  |  |
|  |  | Spock3 |  |  |  |  |
|  |  | Spon1 |  |  |  |  |
|  |  | Stc2 |  |  |  |  |
|  |  | Tcam1 |  |  |  |  |
|  |  | Tfrc |  |  |  |  |
|  |  | Trabd2b |  |  |  |  |
|  |  | Trpc3 |  |  |  |  |
| b |  | Lgr5 |  |  |  |  |
|  |  | Lhb |  |  |  |  |
|  |  | Lpl |  |  |  |  |
|  |  | Ly6c2 |  |  |  |  |
|  |  | Ly9 |  |  |  |  |
|  |  | Mbl2 |  |  |  |  |
|  |  | Mip |  |  |  |  |
|  |  | Ms4a1 |  |  |  |  |
|  |  | Mup12 |  |  |  |  |
|  |  | Ndp |  |  |  |  |
|  |  | Nox4 |  |  |  |  |
|  |  | Npy2r |  |  |  |  |
|  |  | Ntrk1 |  |  |  |  |
|  |  | Olr1 |  |  |  |  |
|  |  | Oosp2 |  |  |  |  |
|  |  | Opn1sw |  |  |  |  |
|  |  | Plxnb1 |  |  |  |  |
|  |  | Prss23 |  |  |  |  |
|  |  | Ptprz1 |  |  |  |  |
|  |  | S1pr3 |  |  |  |  |
|  |  | Serpina3c |  |  |  |  |
|  |  | Serpina9 |  |  |  |  |
|  |  | Serpine2 |  |  |  |  |
|  |  | Sfrp1 |  |  |  |  |
|  |  | Slc2a2 |  |  |  |  |

**Supplemental Table S4.** DEGs categorized in CC terms of GSE207379 and GSE146934.
